# General photonic-crystal slab cavity design for scalable high-power single-mode lasing

**DOI:** 10.1038/s41377-026-02448-6

**Published:** 2026-08-20

**Authors:** Ming Zhou, Mingsen Pan, Chhabindra Gautam, Subhashree Seth, Thomas Rotter, Ganesh Balakrishnan, Weidong Zhou, Shanhui Fan

**Affiliations:** 1 Department of Electrical Engineering, Stanford University, Stanford, CA USA; 2 Department of Electrical Engineering, University of Texas at Arlington, Arlington, TX USA; 3 Department of Electrical and Computer Engineering, University of New Mexico, Albuquerque, NM USA

**Keywords:** Lasers, LEDs and light sources, Photonic crystals

## Abstract

Scaling up the area of photonic-crystal surface-emitting lasers (PCSELs) to produce higher output power while maintaining single-mode operation has been a longstanding objective in photonics and laser physics. Achieving this goal requires two fundamental physics: the difference between the modal losses of the fundamental and higher-order modes, and the mode competition between them. Both physics contribute to the lasing threshold difference between the fundamental and higher-order modes and have been considered individually in past works. But there has not been a theoretical study that treats both physics together for the design of PCSELs. Here we provide a theoretical framework to elucidate the interplay between differential modal losses and mode competition in PCSELs. Based on this framework, we introduce designs based on spatial engineering of photonic-crystal slabs to simultaneously control the modal properties and mode competition. Compared to designs that focus on only one of these physics, our designs can increase the difference between the lasing thresholds of the fundamental and higher-order modes by orders of magnitude. Our findings provide new insights into realizing scalable PCSELs for large-area single-mode lasing.

## Introduction

Realizing high-power single-mode semiconductor surface-emitting lasers has been a long-standing goal in photonics and laser physics, and has motivated significant recent efforts. Standard semiconductor surface-emitting lasers can only sustain single-mode operation with a limited size of emission area and a limited pump power, beyond which the lasers support multiple lasing modes, and the beam quality degrades. Further increasing the emission area or increasing the pumping power to increase the output power inevitably leads to the onset of multimode lasing, limiting the maximum emission power in the single-mode regime^1–3^. To overcome the limitations in standard surface-emitting laser structures, in recent years, many innovative designs based on photonic crystals have been proposed and demonstrated^4–13^.

In general, as the laser emission area increases, the difference in lasing thresholds between the fundamental and higher-order modes decreases, eventually leading to multimode lasing^14^. Thus, one needs to increase the difference in thresholds between the fundamental and higher-order modes to increase the maximum single-mode emission power. There are several approaches to accomplish this goal in the design of the photonic-crystal slab cavity. One widely utilized approach is to increase the differential modal losses between the fundamental and higher-order modes^8,15^. This is typically achieved by tuning the structure of the photonic crystal to control the radiation losses of the fundamental and higher-order modes^7,8,11,12^. By increasing the difference between the radiation losses, the threshold difference can be increased.

Alternatively, the threshold difference can also be increased by controlling the nonlinear modal interactions between the fundamental and higher-order modes, which are determined by their correlation in space^1–3^. In general, when the intensity distribution of the fundamental mode significantly overlaps with that of higher-order modes in space, it can suppress the gain of higher-order modes, making it difficult for them to reach threshold and start lasing. This is known as the gain-clamping effect^1–3,16,17^, and has been observed in wave-chaotic cavities^16,18–21^, where wave-chaotic modes can overlap strongly with each other in space. Recently, ref. ^9,13^ designed photonic-crystal slab structures based on open-Dirac singularities^9^ and artificial gauge fields^13^, where the fundamental mode has a flat spatial profile, resulting in a strong spatial overlap between the fundamental and higher-order modes. Ideally, a perfectly uniform modal profile of the fundamental mode can prevent lasing of higher-order modes for any pump strength^16^. In practice, a fundamental mode that is spatially as uniform as possible can significantly increase the thresholds of higher-order modes.

The physics of differential modal losses and mode competition exists in any semiconductor laser structure. Yet for photonic-crystal surface-emitting lasers (PCSELs), there has not been a theoretical study that takes both physics into consideration for the design of photonic-crystal slab cavities. In this paper, we provide a theoretical framework to elucidate the interplay between differential modal losses and mode competition. Using this framework, we discuss the various design considerations for photonic-crystal slab cavities. We also suggest an alternative cavity design that utilizes both the physics of differential modal loss and mode competition to improve the threshold difference between the fundamental and higher-order modes as the laser structure scales up to a larger area. Our design complements existing designs and has the potential to further increase the threshold difference and single-mode emission area, paving the way for the realization of large-area high-power single-mode lasing.

## Results

### Basics of PCSEL

**Fig. 1:**
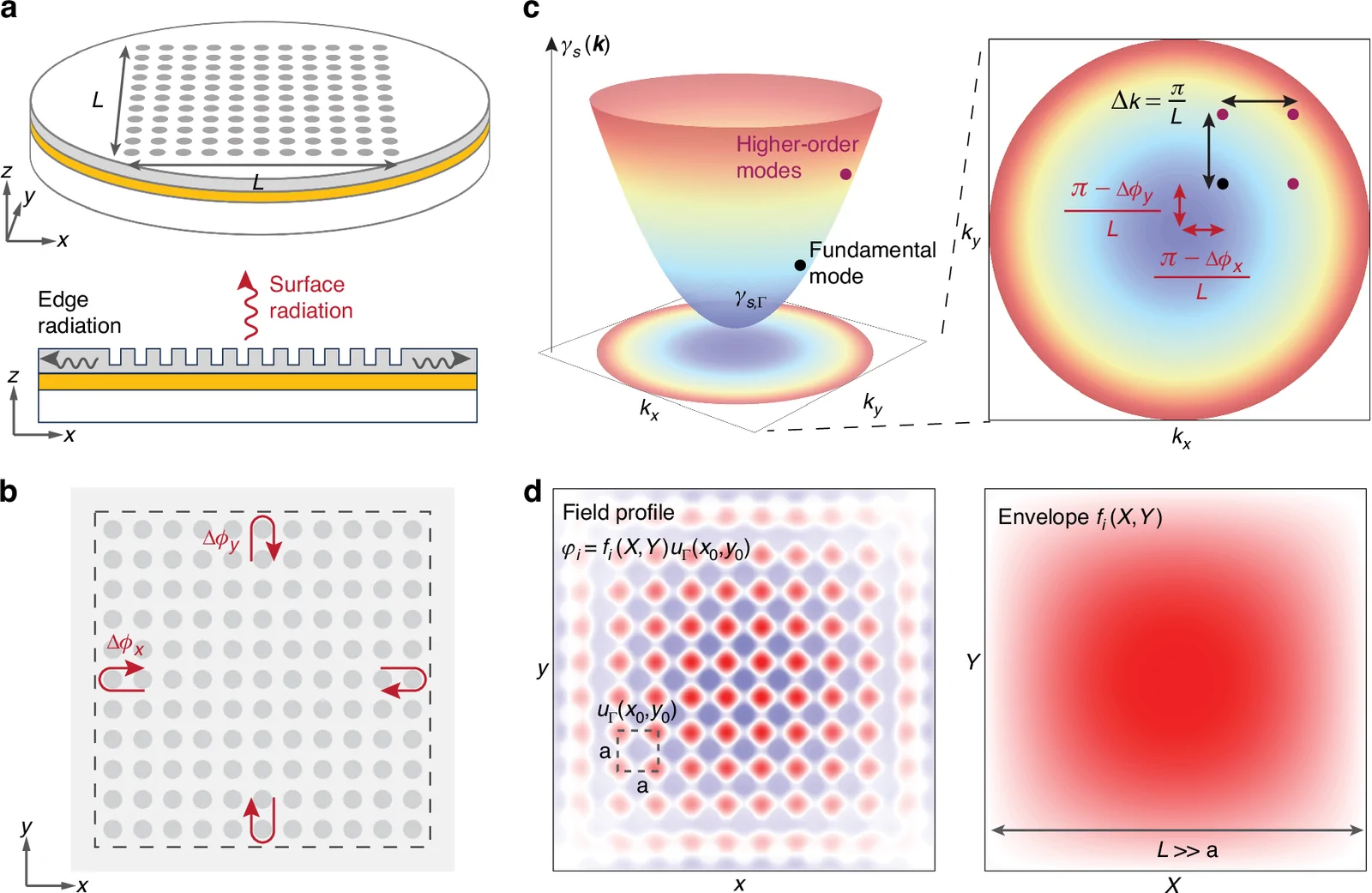
Modal properties of finite-size photonic-crystal surface-emitting lasers (PCSELs). **a** Schematic of finite-size PCSELs. Top: schematic. Bottom: side view and radiation loss mechanisms in PCSEL. **b** Reflection phases at the lateral boundaries of the PCSEL (black dashed line). **c** The radiation constant ${\gamma }_{s}({\boldsymbol{k}})$ as a function of wavevector ${\boldsymbol{k}}$ around ${\mathbf{\Gamma }}$ point. The dots denote the discretized wavevectors ${{\boldsymbol{k}}}_{i}$ of the fundamental and higher-order modes in finite-size PCSELs. The discretized wavevector for the fundamental mode is located away from the Brillouin zone center ${\mathbf{\Gamma }}$ with an offset determined by the reflection phases shown in **b**. The spacing between the wavevectors is determined by the lateral size L. **d** Field profile (left) and envelope profile (right) of a typical fundamental mode within a finite-size PCSEL

Figure 1 shows the schematic of a finite-size PCSEL, which consists of a photonic-crystal slab with spatially uniform lattices. The photonic crystal slab is periodic in the lateral directions, with the same lateral size of L in both x and y directions. In general, in the design of PCSEL, the objective is to achieve single-mode lasing with the emitted beam along the direction normal to the slab, and with the beam profile approximating that of a fundamental Gaussian beam. Moreover, for high-power applications, one aims to achieve such single-mode lasing with a lateral size that is as large as possible. As such, it is important to understand the modal properties of the finite-size PCSEL.

There have been various numerical approaches developed to compute the modal properties of finite-size PCSEL structures, including coupled-wave analysis^14,22^, envelope function approximation (EFA)^23^, and perturbation theory^9^. Complementary to these numerical approaches, here we provide a general analytic theory to illustrate the dependency of the modal properties of PCSEL on photonic band structure. This analytic theory allows us to understand the tradeoff of various considerations in existing designs, and to develop alternative designs of PCSEL for scalable high-power single-mode lasing.

In the analysis of a finite-size PCSEL, one usually starts with the complex band structure of a corresponding photonic-crystal slab structure that is infinitely periodic in the lateral directions. The complex band structure has the form of $\omega \left({\boldsymbol{k}}\right)+i{\gamma }_{s}\left({\boldsymbol{k}}\right)$, where $\omega \left({\boldsymbol{k}}\right)$ and ${\gamma }_{s}\left({\boldsymbol{k}}\right)$ are the angular frequency and radiation rate of a mode of the infinite structure at the in-plane wavevector ${\boldsymbol{k}}$. In general, since one aims to achieve emissions along the normal direction, the relevant part of the band structure is near ${\mathbf{\Gamma }}$ at the center of the first Brillouin zone with ${\boldsymbol{k}}={\boldsymbol{0}}$. Therefore, it is usually sufficient to perform an expansion of the band structure around ${\mathbf{\Gamma }}$. We can assume both $\omega \left({\boldsymbol{k}}\right)$ and ${\gamma }_{s}\left({\boldsymbol{k}}\right)$ exhibit power-law dependence on ${\boldsymbol{k}}$ around ${\mathbf{\Gamma }}$, in the form of

1 $$\omega \left({\boldsymbol{k}}\right)={\omega }_{{\mathbf{\Gamma }}}+A{\left|{\boldsymbol{k}}\right|}^{l}$$

2 $${\gamma }_{s}\left({\boldsymbol{k}}\right)={\gamma }_{s,{\mathbf{\Gamma }}}+\,{C}_{s}{\left|{\boldsymbol{k}}\right|}^{s}$$

where the coefficients A and ${C}_{s}$ are determined by the specific unit-cell structure. As a few examples, l=2 and s=2 for quadratic band edge at **Γ**^8^, and l=1 and s=2 for linear dispersions such as Dirac cone at **Γ**^9,24^.

One can then infer the modal properties in the finite-size structure using the information of the band structure of the infinite photonic-crystal slab. For a finite-size structure with lateral size L along both x and y directions (Fig. 1a), the in-plane wavevectors of the modes satisfy the following condition

3 $${\boldsymbol{k}}=\left(\left(m+1\right)\frac{\pi }{L}-\frac{\Delta {\phi }_{x}}{L},\left(n+1\right)\frac{\pi }{L}-\frac{\Delta {\phi }_{y}}{L}\right),\,m,n=0,1,2,\ldots$$

where $\Delta {\phi }_{x}$ and $\Delta {\phi }_{y}$ are the reflection phases at the lateral boundaries (Fig. 1b). The fundamental mode corresponds to m=0 and n=0, with an in-plane wavevector of ${{\boldsymbol{k}}}_{0}=\left(\frac{\pi -\Delta {\phi }_{x}}{L},\frac{\pi -\Delta {\phi }_{y}}{L}\right)$. There are two doubly degenerate first-order modes corresponding to m=0 and n=1, and m=1 and n=0. For simplicity, we consider the mode with in-plane wavevector$\,{{\boldsymbol{k}}}_{1}=\left(\frac{2\pi -\Delta {\phi }_{x}}{L},\frac{\pi -\Delta {\phi }_{y}}{L}\right)$ as the first-order mode throughout our discussion. The mode profile of the ${i}^{{th}}$ mode can be approximated using multiple-scale expansion^23,25,26^ as

4 $${\varphi }_{i}\left({\boldsymbol{r}}\right)={u}_{{\mathbf{\Gamma }}}\left({x}_{0},{y}_{0},z\right){f}_{i}\left(X,Y\right)$$

5 $${f}_{i}\left(X,Y\right)\approx \left({e}^{i{{\boldsymbol{k}}}_{i}^{x}X}+{\left(-1\right)}^{i}{e}^{-i{{\boldsymbol{k}}}_{i}^{x}X}\right)\left({e}^{i{{\boldsymbol{k}}}_{i}^{y}Y}+{e}^{-i{{\boldsymbol{k}}}_{i}^{y}Y}\right)$$

where ${u}_{{\mathbf{\Gamma }}}\left({x}_{0},{y}_{0},z\right)$ is the Bloch mode at ${\mathbf{\Gamma }}$. ${f}_{i}\left(X,Y\right)$ is the lateral modal envelope function that modulates the Bloch mode. ${{\boldsymbol{k}}}_{i}^{x}$ and ${{\boldsymbol{k}}}_{i}^{y}$ are the x- and y-components of the in-plane wavevector ${{\boldsymbol{k}}}_{i}$. Here $({x}_{0},{y}_{0})$ and (X,Y) describe the small scale within each unit cell and the large scale across the entire photonic-crystal slab, respectively. An example of the mode profile and its corresponding envelope is shown in Fig. 1d.

There are two radiative loss mechanisms for the modes in a finite-size photonic-crystal slab, the surface radiation loss along the direction normal to the slab and the edge radiation along the lateral directions (Fig. 1a, middle). The surface radiation loss of the ${i}^{{th}}$ mode is approximated by substituting the wavevector ${{\boldsymbol{k}}}_{i} \sim {L}^{-1}$ into Eq. (2), which leads to ${\alpha }_{s,i}={\gamma }_{s,\Gamma }+{C}_{s,i}{L}^{-s}$. For the edge radiation loss, there are two radiation mechanisms. One mechanism is the direct transmission through the edge interface. Its loss scales as ${C}_{e,i}^{{tr}}{L}^{-l}$, as can be obtained by considering the group velocity of the mode, with ${C}_{e,i}^{{tr}}$ being a coefficient related to the group velocity. The other mechanism is the scattering to all other directions. It is proportional to the ratio between the energy at the edge and within the cavity, which scales as

6 $${\alpha }_{e,i}^{{sc}} \sim \frac{\iint {\left|{\varphi }_{i}\left({{\boldsymbol{r}}}_{{edge}}\right)\right|}^{2}d{\boldsymbol{r}}}{\iint {\left|{\varphi }_{i}\left({\boldsymbol{r}}\right)\right|}^{2}d{\boldsymbol{r}}} \sim {L}^{-1}$$

Here we use the fact that energy in the cavity scales with the cavity area as ${L}^{2}$, while the energy at the edge scales with the circumference as ${L}$. By lumping both mechanisms together, the total modal loss of the ${i}^{{th}}$ mode can be written as

7 $${\alpha }_{i}={\gamma }_{s,\Gamma }+{C}_{s,i}{L}^{-s}+{C}_{e,i}^{{sc}}{L}^{-1}+{C}_{e,i}^{{tr}}{L}^{-l}$$

where ${C}_{e,i}^{{sc}}$ is a coefficient related to the field amplitude ${\varphi }_{i}\left({{\boldsymbol{r}}}_{{edge}}\right)$ at the edge. For most conventional PCSEL cavities, ${\varphi }_{i}\left({{\boldsymbol{r}}}_{{edge}}\right)\approx 0$ and thus ${C}_{e,i}^{{sc}}\approx 0$. Equation (7) agrees well with existing literature, which considers either quadratic band edge^8^ with ${C}_{e,i}^{{sc}}\approx 0$, or linear dispersion^9,24^.

To achieve single-mode lasing in the fundamental mode, the fundamental mode should have the lowest modal loss among all modes, i.e., ${\alpha }_{0} < {\alpha }_{i\ne 0}$. Typically, for a structure that is sufficiently large, the field amplitude at the edge can be weak, therefore the rate of surface radiation dominates over that of the radiation from the edge of the photonic crystal, i.e., ${\alpha }_{s,i}\gg \,{\alpha }_{e,i}$. In this case, the surface radiation is the dominant component in the radiative loss, hence a minimum at ${\mathbf{\Gamma }}$ in the ${\gamma }_{s}\left({\boldsymbol{k}}\right)$ dispersion (Fig. 1c) is sufficient to ensure that the fundamental mode has the lowest loss and is the first mode to lase. For more general design of cavities, both the surface radiation rate and the edge radiation rate may contribute significantly to the total radiation loss. Consequently, the coefficients$\,{\gamma }_{s,\Gamma }$, ${C}_{s}$, ${C}_{e,i}$, and the scaling exponents s and l generally need to be optimized such that ${\alpha }_{0} < {\alpha }_{i\ne 0}$. We will discuss strategies to achieve this condition in the following sections.

Once the condition of ${\alpha }_{0} < {\alpha }_{i\ne 0}$ is satisfied, the goal then is to maintain single-mode lasing at a pumping strength that is as high as possible. In general, beyond the threshold ${g}_{0}={\alpha }_{0}$ of lasing for the fundamental mode, the lasing remains in the fundamental mode until the first-order mode starts to lase at a higher threshold ${g}_{1}$. The maximum output power in the single-mode regime is controlled by the difference $\triangle {g}_{{th}}={g}_{1}-{g}_{0}$ between these two thresholds. Because of mode competition, the threshold ${g}_{1}$ for the first-order mode deviates from its modal loss rate *α*_1_^2,3^, due to the effect of mode competition, as we will analyze in the next section.

### Review of mode competition in multimode cavities

In the semiclassical laser theory framework, the lasing dynamics are described by the Maxwell–Bloch equations, which are a set of nonlinear coupled differential equations. However, numerically solving the Maxwell–Bloch equations for the steady-state lasing modes in PCSELs is computationally expensive due to the large area of PCSELs. Here we adopt the well-known near-threshold approximation to qualitatively illustrate the lasing dynamics^2^. More quantitatively accurate dynamics above the threshold can be obtained by using well-developed steady-state ab initio laser theories^16,27–32^ (SALT), which do not assume the near-threshold approximation. Nevertheless, the physics of gain clamping remains the same in both the simplest lasing model considered here and SALT.

The fundamental physics for the threshold difference between the fundamental and first-order modes in a PCSEL can be illustrated using the following equations of two-mode lasing^3^:

8 $$\frac{d}{{dt}}{I}_{0}=2{I}_{0}\left(g-{\alpha }_{0}-{\chi }_{00}{I}_{0}-{\chi }_{01}{I}_{1}\right)$$

9 $$\frac{d}{{dt}}{I}_{1}=2{I}_{1}\left(g-{\alpha }_{1}-{\chi }_{10}{I}_{0}-{\chi }_{11}{I}_{1}\right)$$

10 $${\chi }_{{ij}}=\int d{\boldsymbol{r}}G({\boldsymbol{r}}){\varphi }_{i}^{2}\left({\boldsymbol{r}}\right){\left|{\varphi }_{j}^{2}\left({\boldsymbol{r}}\right)\right|}^{2}$$

where ${I}_{i}$ and ${\varphi }_{i}({\boldsymbol{r}})$ are the output intensity and normalized mode profile of the ${i}^{{th}}$ mode, respectively. The interaction coefficient ${\chi }_{{ij}}$ defines the nonlinear modal interaction between the ${i}^{{th}}$ and ${j}^{{th}}$ mode, where $G({\boldsymbol{r}})$ is a fixed pump profile. Throughout our discussions, we assume $G\left({\boldsymbol{r}}\right)=1$, which corresponds to uniform pumping within the cavity. Note that ${\chi }_{{ij}}$ is complex as photonic-crystal slabs are typically open systems^27^. However, the imaginary part of ${\chi }_{{ij}}$ is negligible for the high-Q modes in photonic-crystal slabs, and thus we assume ${\chi }_{{ij}}\approx \mathrm{Re}\left({\chi }_{{ij}}\right)$. At large lateral size L, the free spectral range of the cavity is small. Therefore, we can ignore the frequency dependence of the gain spectrum and assume the two modes have the same unsaturated gain g. By performing stability analysis around the steady-state single-mode solution, i.e., ${I}_{0} > 0$ and ${I}_{1}=0$, we can obtain the threshold difference between the fundamental and first-order modes as

11 $$\Delta {g}_{{th}}=\frac{\Delta \alpha }{1-{\mathcal{F}}}$$

where

12 $${\mathcal{F}}=\frac{\int d{\boldsymbol{r}}G\left({\boldsymbol{r}}\right){\varphi }_{1}^{2}\left({\boldsymbol{r}}\right){\left|{\varphi }_{0}(r)\right|}^{2}}{\int d{\boldsymbol{r}}G\left({\boldsymbol{r}}\right){\varphi }_{0}^{2}\left({\boldsymbol{r}}\right){\left|{\varphi }_{0}(r)\right|}^{2}}$$

Here $\Delta \alpha ={\alpha }_{1}-{\alpha }_{0}$ and ${\mathcal{F}}$ are the differential modal loss and the spatial overlap factor between the fundamental and first-order modes, respectively. Equation 11 qualitatively agrees with the threshold difference derived using SALT^16^, which also predicts perfect gain clamping with complete spatial overlap and ${\mathcal{F}}=1$. According to Eq. 11, to increase the threshold difference between the fundamental and first-order modes, the keys are to increase the differential modal loss and the spatial overlap between the modes.

### Scaling challenge of differential modal loss in PCSEL

While a large differential modal loss is always desired, the scaling of differential modal loss in finite-size photonic crystals makes it challenging to achieve this objective. As the PCSEL emission area increases, the spacing between the fundamental and first-order modes in the momentum space becomes smaller, as shown in Fig. 1c, leading to smaller differential modal loss. For PCSELs with lateral size of L in both x and y directions, the scaling of differential modal loss $\Delta \alpha$ can be obtained from Eq. 7 as

13 $$\Delta \alpha =\Delta {C}_{e}^{{sc}}{L}^{-1}+\Delta {C}_{e}^{{tr}}{L}^{-l}+\Delta {C}_{s}{L}^{-s}$$

Here we define $\Delta {C}_{e}^{{sc}}={C}_{e,1}^{{sc}}-{C}_{e,0}^{{sc}}$, $\Delta {C}_{e}^{{tr}}={C}_{e,1}^{{tr}}-{C}_{e,0}^{{tr}}$ and $\Delta {C}_{s}={C}_{s,1}-{C}_{s,0}$ for compactness.

Therefore, the differential modal loss diminishes as the lateral size L increases, making it challenging to maintain threshold difference for large systems. Moreover, a minimum differential modal loss $\Delta {\alpha }_{c}$ is often required in practice such that single-mode lasing can be maintained without stringent control over the pumping power^7,8^. As such, single-mode lasing can only be sustained up to a maximum lateral size ${L}_{c}$ such that $\Delta \alpha > \Delta {\alpha }_{c}$. For a particular PCSEL design, the large-L behavior of the differential modal loss is governed by the leading term in Eq. 13, ${\Delta}a\,{\sim}\,{\Delta}{CL}^{-{{p}}}$, where p is the leading scaling exponent and $\Delta{C}$ is the corresponding coefficient.

The scaling of differential modal loss thus directly affects the maximum lateral size ${L}_{c}$. A smaller scaling exponent, e.g., p=1, causes the differential modal loss to decrease more slowly with lateral size and therefore allows the minimum required differential modal loss to be maintained up to a larger lateral size. This behavior is favorable for scalable high-power single-mode lasing. In existing PCSEL designs, such a favorable scaling exponent p=1 has only been realized in linear dispersion with l=1. For most existing designs based on quadratic dispersion, the linear term $\Delta {C}_{e}^{{sc}}{L}^{-1}$ in the differential modal loss vanishes due to the weak field amplitude at the edges of the PCSEL cavity. However, it is important to note that the vanishing field at the edges of the cavity is not intrinsic to quadratic dispersion. As we will show later, the field amplitude at the lateral boundaries is determined by the reflection phases $\Delta {\phi }_{x}$ and $\Delta {\phi }_{y}$ at the interface. Thus, it is possible to obtain the favorable scaling exponent p=1 in any photonic dispersion.

### Threshold enhancement due to spatial overlap

In general, the threshold difference is proportional to the differential modal loss, with a gain-enhancement factor of $\frac{1}{1-{\mathcal{F}}}$ resulting from the spatial overlap between the fundamental and first-order modes. The gain-enhancement factor can drastically increase the threshold difference by orders of magnitude when ${\mathcal{F}}$ approaches unity. In the ideal case of complete spatial overlap between the fundamental and higher-order modes, i.e., ${\mathcal{F}}=1$, the threshold difference diverges, and single-mode lasing is guaranteed regardless of the pumping power. Even when the spatial overlap is not perfect, as is typical in practice due to fabrication imperfections, it still can lead to giant enhancement factors, e.g., ${\mathcal{F}}=0.99$ corresponds to an enhancement factor of 100. This effect has been leveraged to achieve single-mode lasing in wave-chaotic cavities, where the random spatial intensity distributions of the modes strongly overlap with each other^16,18–21^.

However, without specific engineering of the reflection phases $\Delta {\phi }_{x}$ and $\Delta {\phi }_{y}$ at the lateral boundaries (Fig. 1b), the spatial overlap factor between the modes in a PCSEL cavity is typically well below unity. For most photonic-crystal slabs with spatially uniform lattices, $\Delta {\phi }_{x}=0$ and $\Delta {\phi }_{y}=0$, resulting in ${k}_{0}=\left(\frac{\pi }{L},\frac{\pi }{L}\,\right)$ for the fundamental mode and ${{\boldsymbol{k}}}_{1}=\left(\frac{2\pi }{L},\frac{\pi }{L}\right)$ for the first-order mode. The corresponding mode profiles and the spatial overlap factor can then be calculated using Eq. 5 and Eq. 12, and one finds that ${\mathcal{F}}=\frac{2}{3}$. In stark contrast, when the reflection phases are specifically engineered to satisfy $\Delta {\phi }_{x}=\pi$ and $\Delta {\phi }_{y}=\pi$, the wavevector ${{\boldsymbol{k}}}_{0}$ of the zeroth mode shifts to the Brillouin zone center ${\mathbf{\Gamma }}$ (Fig. 1c). The field envelope of the zeroth mode ${f}_{0}$ thus becomes spatially uniform and completely overlaps with that of all higher-order modes, resulting in ideal spatial overlap factor ${\mathcal{F}}=1$. In other words, one can significantly increase the spatial overlap by carefully engineering the lateral boundaries of the finite-size photonic-crystal slab^13^.

### Lasing mode selection and surface-emission efficiency

While controlling the scaling of differential modal loss and the field envelopes can drastically increase the threshold difference, they may lead to undesired consequences. First, the mode with the desired field profile may not be the mode with the lowest loss if the field envelopes are drastically modified. Second, even if the mode with the desired field profile remains as the mode with the lowest loss and threshold, its surface-emission efficiency ${\eta }_{{SE}}$, an important laser performance metric, can have undesired scaling. For a PCSEL of lateral size L, the surface-emission efficiency ${\eta }_{{SE}}$ of the fundamental mode with wave vector ${k}_{0}=\left(\frac{\pi }{L},\frac{\pi }{L}\,\right)$ is given by

14 $${\eta }_{{SE}}=\frac{{\gamma }_{s,{\mathbf{\Gamma }}}+{C}_{s,0}{L}^{-s}}{{\gamma }_{s,{\mathbf{\Gamma }}}+{C}_{s,0}{L}^{-s}+{C}_{e,0}^{{tr}}{L}^{-l}+{C}_{e,0}^{{sc}}{L}^{-1}+{\alpha }_{{in}}}$$

Here ${\alpha }_{{in}}$ is the intrinsic material loss. Therefore, to achieve high surface-emission efficiency, the surface radiation loss ${\gamma }_{s,{\mathbf{\Gamma }}}+{C}_{s}{L}^{-s}$ needs to be at least comparable, and preferably larger than the combination of the edge radiation losses and the intrinsic loss, i.e., ${\alpha }_{{in}}+{C}_{e,0}^{{tr}}{L}^{-l}+{C}_{e,0}^{{sc}}{L}^{-1}$. For this reason, it is crucial to make both the radiation constant ${\gamma }_{s,{\mathbf{\Gamma }}}$ and the curvature of the ${\gamma }_{s}({\boldsymbol{k}})$ dispersion sufficiently large. On the other hand, the coefficients ${C}_{e,0}^{{tr}}$ and ${C}_{e,0}^{{sc}}$ should be minimized. Combining these conditions together ensures that the radiation from the modes is dominated by their surface radiation, and the surface-emission efficiency is high. Meanwhile, under these conditions, a minimum at ${\mathbf{\Gamma }}$ in the ${\gamma }_{s}\left({\boldsymbol{k}}\right)$ dispersion (Fig. 1c) is sufficient to ensure that the fundamental mode is the first mode to lase.

However, modifying the scaling of differential modal loss and the field envelopes can break both conditions mentioned above. In the case of spatially uniform fundamental modes, the high field amplitude at the lateral boundaries makes it difficult to minimize the coefficient $C^{(sc)}_{e,0}$. Furthermore, if there exist bound states in the continuum (BICs) at ${\mathbf{\Gamma }}$, ${\gamma }_{s,{\boldsymbol{\Gamma }}}=0$^9,11,13,24^ and the surface-emission efficiency can be low, especially when the dispersion is linear and the edge radiation loss scales linearly with L, i.e., l=1. Both trade-offs can be overcome by specifically engineering the lateral boundaries of the photonic-crystal structure, as we will show in the following sections.

### Design considerations for scalable PCSEL design

We can now summarize the conditions required to achieve a PCSEL design that can be scaled up to a large emission area and a high pumping power, while maintaining single-mode lasing. To achieve this goal, the PCSEL design needs to satisfy the following criteria:

1. Favorable scaling of differential modal loss, e.g., linear scaling $\Delta \alpha \sim {L}^{-1}$, such that single-mode operation can be sustained up to a larger emission area.
2. A fundamental mode envelope ${f}_{0}\left(X,Y\right)$ that is as spatially uniform as possible, such that the modal overlap approaches unity, i.e., ${\mathcal{F}}{\mathscr{\to }}1$., and the threshold difference is enhanced by orders of magnitude due to mode competition.
3. In addition to satisfying the above two conditions, the photonic-crystal structures should still be able to achieve sufficiently large ${\gamma }_{s,{\mathbf{\Gamma }}}$ and ${C}_{s}$, and minimized ${C}_{e,i}$, such that the fundamental mode lases at the lowest threshold and surface-emission efficiency can be kept high.

### Existing PCSEL designs

**Fig. 2:**
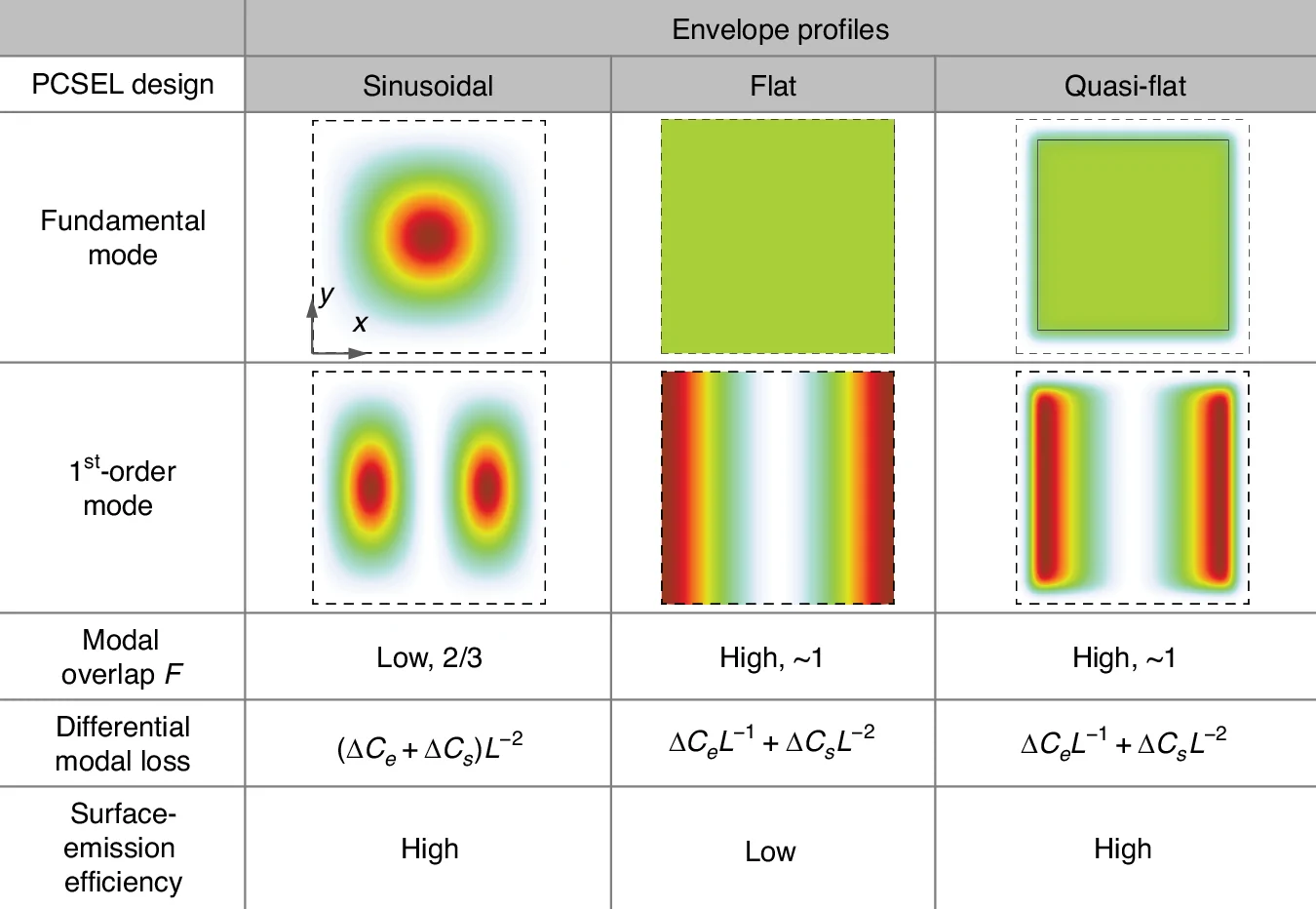
Modal intensity profiles and scaling laws in the sinusoidal cavity, the flat cavity, and our quasi-flat cavity. The sinusoidal cavity exhibits sinusoidal modal profiles, quadratic scaling of threshold difference, and relatively low modal overlap factor, but its surface-emission efficiency can be kept high. The flat cavity exhibits a completely flat modal profile for the fundamental mode, linear scaling of threshold difference, and a high modal overlap factor, but its surface-emission efficiency is low. In contrast, our quasi-flat cavity combines the excellent characteristics of both sinusoidal and flat cavities, i.e., high modal overlap, linear scaling of threshold difference, and high surface-emission efficiency

With the above design considerations, we briefly review existing PCSEL designs and their trade-offs. Existing PCSEL designs typically exhibit two different dispersions around ${\mathbf{\Gamma }}$, quadratic and linear dispersions. Figure 2 summarizes the modal properties and lasing performance metrics of these designs. For PCSEL designs with quadratic dispersions^6,7,11^, the field envelopes of the zeroth and first-order modes resemble sinusoidal waves (top two rows in Fig. 2, left column), and the field amplitudes at the lateral boundaries (dashed rectangles) are minimized. We refer to PCSEL designs with such field envelopes as the sinusoidal PCSEL design. Because of the low amplitude at the lateral boundaries, the edge radiation in the sinusoidal PCSEL design is minimized. Meanwhile, for most photonic crystals with quadratic dispersions, ${\gamma }_{s,{\mathbf{\Gamma }}}$ and the curvature ${C}_{s}$ in the ${\gamma }_{s}({\boldsymbol{k}})$ dispersion can be readily controlled by tuning the unit-cell structure. Consequently, the surface emission efficiency of the sinusoidal design is typically high. However, as we discussed earlier for quadratic dispersions, the scaling of the differential modal loss in the sinusoidal PCSEL design is quadratic, scaling as $\Delta \alpha =\left(\Delta {C}_{e}+\Delta {C}_{s}\right){L}^{-2}$. Furthermore, the spatial overlap factor in the sinusoidal design is only ${\mathcal{F}}=\frac{2}{3}$. While scalable ultra-large-area single-mode lasing has been demonstrated using the sinusoidal PCSEL design^6,7,11^, improving both the scaling of differential modal loss and the spatial overlap can further increase the lasing emission area and output power, potentially by orders of magnitude.

In the case of PCSEL designs with linear dispersions^9^, owing to the zero effective refractive index at ${\mathbf{\Gamma }}$, the field envelope of the fundamental mode ${f}_{0}$ is spatially uniform as shown in Fig. 2. We refer to PCSEL designs with such a fundamental mode as the flat PCSEL design. It possesses an ideal spatial overlap factor ${\mathcal{F}}=1$ and linear scaling of differential modal loss, which scales as $\Delta \alpha =\Delta {C}_{e}{L}^{-1}+\Delta {C}_{s}{L}^{-2}$. However, as we discussed earlier, the surface-emission efficiency in the flat PCSEL design is low due to the existence of BIC at ${\mathbf{\Gamma }}$ and ${\gamma }_{s,{\mathbf{\Gamma }}}=0$. While it is possible to enhance the surface-emission efficiency by introducing perturbations in the unit-cell structure of the photonic crystal to break the BIC and increase ${\gamma }_{s,{\mathbf{\Gamma }}}$, because existing demonstration employs accidental degeneracy at ${\mathbf{\Gamma }}$ to realize the linear dispersion^9^, such perturbations can lift this accidental degeneracy and break the linear dispersion^33^. As a result, the scaling of $\Delta \alpha$ is no longer linear and the fundamental mode is no longer spatially uniform.

### Quasi-flat PCSEL design

To circumvent the trade-offs in the sinusoidal and the flat PCSEL designs while preserving their respective advantages, we propose the quasi-flat PCSEL design. To retain the high surface-emission efficiency in the sinusoidal PCSEL design, our design is also based on a photonic crystal with quadratic dispersions. Unlike existing designs that typically consist of spatially uniform lattices, our design consists of a spatially uniform bulk region with gain (solid rectangles), which is surrounded by a spatially nonuniform cladding region without gain. The lateral size ${L}_{c}$ of the cladding region is fixed, while the lateral size L of the bulk region can vary. This design allows us to realize unique field envelopes. The claddings can be engineered such that the reflection phases at the bulk-cladding interface satisfy $\Delta {\phi }_{x}=\pi$ and $\Delta {\phi }_{y}=\pi$. As such, the field envelope of the fundamental mode is flat within the bulk region, resulting in an ideal spatial overlap factor ${\mathscr{F}}=1$.

Moreover, the field at the bulk-cladding interface now does not vanish, retaining the favorable linear term $\Delta {C}_{e}^{{sc}}{L}^{-1}$ in the differential modal loss, which otherwise would vanish in conventional designs based on quadratic dispersion. On the other hand, the claddings are engineered so that the field amplitude adiabatically decreases to zero at the lateral boundaries, allowing us to carefully control the edge radiation losses and surface-emission efficiency. In summary, our quasi-flat design opens a potential pathway to simultaneously achieve the required conditions to further scale up the emission area and power of PCSELs, i.e., favorable scaling of $\Delta \alpha$, ideal spatial overlap, and high surface-emission efficiency.

**Fig. 3:**
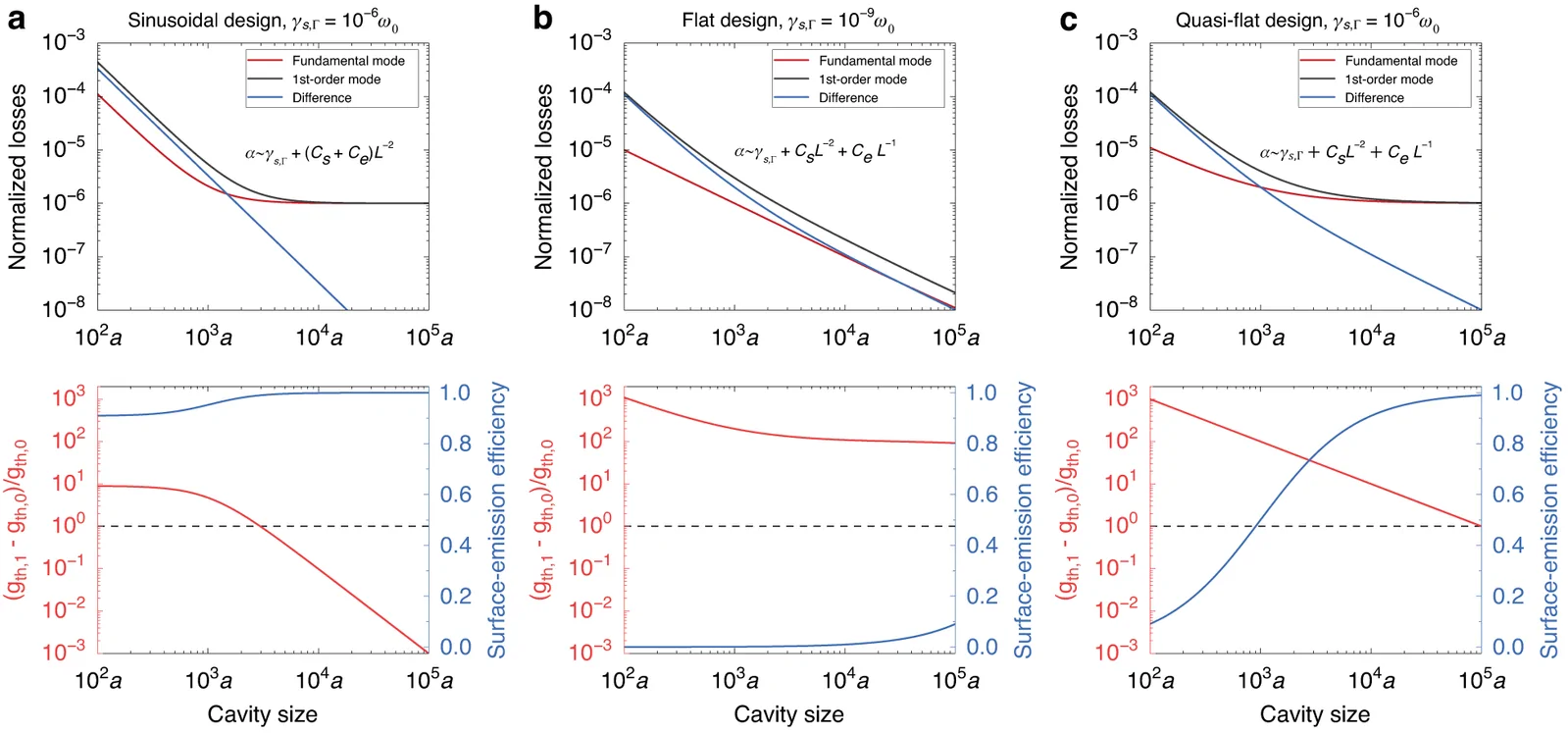
Scaling laws in different designs of PCSELs. Scaling laws in **a** the sinusoidal design, **b** the flat design, and **c** the quasi-flat design. Top row: normalized radiation losses of the fundamental (red) and first-order (black) modes, and the difference between them (blue). Bottom row: normalized threshold difference (red) and surface-emission efficiency (blue). The black dashed line denotes pumping at 2 times of the fundamental threshold

To quantitatively compare the performance of all three designs, we calculate the scaling of normalized threshold differences $\Delta {g}_{{th}}/{g}_{{th},0}$ and surface emission efficiency $\eta ={\alpha }_{s,0}/\left({\alpha }_{s,0}+{\alpha }_{e,0}\right)$ and plot the results in Fig. 3. Here ${g}_{{th},0}$ is the fundamental threshold. For the sinusoidal design with quadratic scaling of $\Delta \alpha \sim {L}^{2}$, we assume the radiation rate of the photonic crystal at the ${\mathbf{\Gamma }}$ point is ${\gamma }_{s,\Gamma }={10}^{-6}{\omega }_{0}$, where ${\omega }_{0}$ is the resonant frequency of the fundamental mode. For lateral sizes ranging from ${10}^{2}a$ to ${10}^{5}a$, where a is the lattice constant of the photonic crystal, the surface-emission efficiency η remains above 90% as shown in Fig. 3a (blue solid line). It is important to note that we only consider the surface and edge radiation losses in our calculations. The actual surface-emission efficiency will be lower due to the presence of the intrinsic material loss ${\alpha }_{{in}}$. Assuming that single-mode operation can only be maintained when the threshold difference $\Delta {g}_{{th}}$ is the same as the fundamental threshold ${g}_{{th},0}$ (black dashed line), the sinusoidal design can reach up to a maximum lateral size of ${{10}^{3} \sim 10}^{4}a$, comparable with the state-of-the-art demonstrations of sinusoidal PCSEL designs^11^. Beyond ${10}^{4}a$, single-mode operation is only achievable slightly above the fundamental threshold due to the shrinking threshold difference, i.e., $\Delta {g}_{{th}}\ll {g}_{{th},0}$, which is not desirable for high-power single-mode operation.

In the case of the flat PCSEL design with $\Delta \alpha \sim {L}^{-1}$, we set ${\gamma }_{s,\Gamma }^{{Dirac}}={10}^{-9}{\omega }_{0}$ and ${\mathcal{F}}=0.99$, assuming the BIC at the ${\mathbf{\Gamma }}$ point is slightly perturbed while preserving a nearly flat fundamental mode. Figure 3b shows the corresponding threshold difference $\Delta {g}_{{th}}$ and surface-emission efficiency *η*. Because of the nearly flat modal profile, the threshold difference can remain 10^2^ times above the fundamental threshold even up to ${10}^{5}a$. Similar scaling for the difference between the quality factors of the fundamental and first-order modes has been reported in ref. ^8^. However, the surface-emission efficiency of such a PCSEL design is almost zero for all lateral sizes ranging from ${10}^{2}a$ to ${10}^{4}a$. It only reaches ~10% when the edge radiation loss ${\alpha }_{e,0} \sim {L}^{-1}$ is eventually comparable with$\,{\gamma }_{s,{\mathbf{\Gamma }}}$.

In stark contrast, our quasi-flat design can simultaneously achieve large threshold differences and high surface-emission efficiencies as shown on Fig. 3c. Here, we assume the quasi-flat design has the same scaling of radiation loss as the flat design, except we set ${\gamma }_{s,\Gamma }={10}^{-6}{\omega }_{0}$, the same as the sinusoidal design. Compared to the flat design, the surface-emission efficiency of our quasi-flat design increases quickly with increasing lateral size L, reaching above 50% once the lateral size is beyond $\sim {10}^{3}a$. Furthermore, the threshold difference of our quasi-flat design is orders-of-magnitude higher than that of the sinusoidal design for all lateral sizes. It remains above the fundamental threshold at lateral size of ${10}^{5}a$ with almost 100% surface-emission efficiency.

Below, we will provide numerical validation of the theoretical discussions above. Our theoretical discussion on the flat design is consistent with the numerical results of ref. ^8^. Therefore, in the numerical validation we will focus only on the quasi-flat cavity design based on quadratic dispersion, and we will contrast such design with the sinusoidal design that is also based on quadratic dispersion.

### Numerical validation of quasi-flat mode envelopes and the scaling laws

**Fig. 4:**
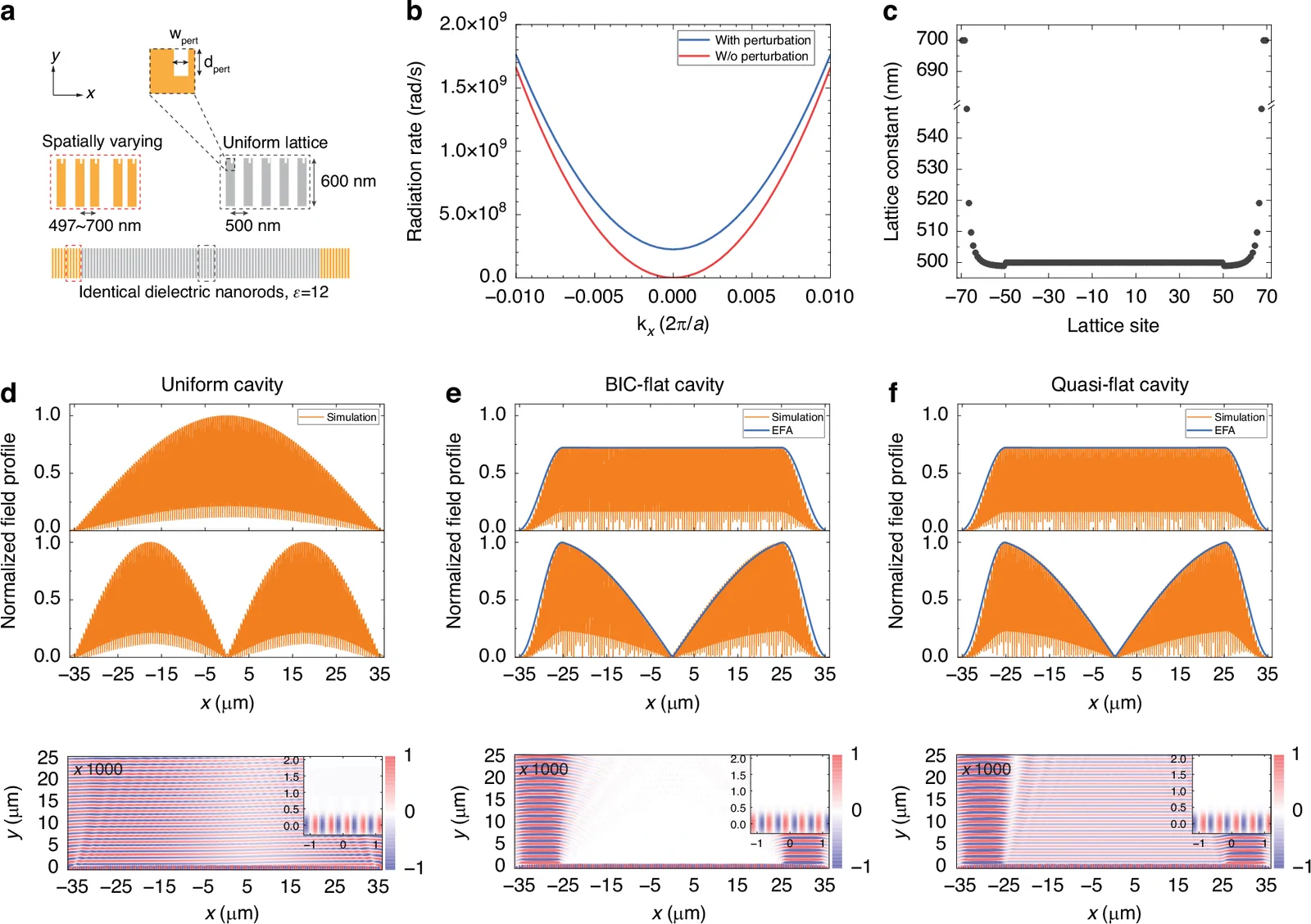
Demonstration of quasi-flat mode in 1D photonic crystal. **a** Schematic of the 1D photonic crystal. It consists of an array of dielectric nanorods with a dielectric constant of 12. The dimensions of the nanorods are $600{nm}$ by $287.5{nm}$. A symmetry-breaking air void is added to the top of the nanorod to perturb the BIC at the ${\mathbf{\Gamma }}$ point. The dimensions of the air void are ${w}_{{pert}}=20{nm}$ and ${d}_{{pert}}=20{nm}$. The lattice constant in the uniform bulk region (grey rods) is $500{nm}$, and varies spatially in the lateral claddings (orange rods). **b** Normalized radiation rate of laterally infinite 1D photonic crystal with and without the small air voids. **c** Spatially varying lattice constants. **d**–**f** Top row: horizontal cut of the electric field distributions (${E}_{z}$) at y=0 for the fundamental mode (top) and the first-order mode in the uniform cavity (**d**), the BIC-flat cavity (**e**), and the quasi-flat cavity (**f**). Bottom row: two-dimensional electric field distribution of the fundamental mode in different cavities. Inset: field distribution within the photonic crystal. The field is normalized by the maximum of the field within the photonic crystal. To clearly visualize the radiation field in air, which is much smaller than the field within the photonic crystal, we scale up the radiation field outside the photonic crystal by 1000 times

We directly validate the above scaling laws in full-wave simulations. Because full-wave simulation of photonic-crystal slabs in three-dimensional (3D) space is computationally expensive for lateral sizes above L~100a, here we consider finite-size gratings in two-dimensional (2D) space to enable us to probe larger lateral sizes and demonstrate scaling behaviors. Figure 4a shows the schematic of the finite-sized grating, which consists of an array of dielectric nanorods with a dielectric constant of 12. The height and the width of the nanorods are $600{nm}$ and $287.5{nm}$, respectively. For a uniform and laterally infinite grating with a lattice constant of $500{nm}$, it supports a quadratic band edge for modes with transverse magnetic polarization with a band edge frequency of $\sim 212{THz}$. It also exhibits a BIC at the ${\mathbf{\Gamma }}$ point due to a symmetrical unit cell structure. To perturb the BIC, we introduce small air voids at the top of the nanorods to break the symmetry, as shown in Fig. 4a. Figure 4b shows the radiation losses corresponding to different wavevectors with and without perturbations, which are normalized by the band edge frequency.

Three different cavities are considered: the spatially uniform cavity with symmetry-breaking air voids, the BIC-flat cavity with spatially varying claddings but without symmetry-breaking air voids, and our quasi-flat design with symmetry-breaking air voids and spatially varying claddings. The spatially varying lattice constants (Fig. 4c) are obtained using the EFA approach we recently developed^23^. Note that the BIC-flat cavity and our quasi-flat cavity are identical except for the symmetry-breaking air voids. Details of the EFA approach, the design procedure, and the spatially varying lattice constants are provided in the Supplementary Information Section 2. For all designs, the total number of nanorods is 140. In the case of BIC-flat and quasi-flat designs, the cavities consist of a uniform bulk region of 100 nanorods, which are surrounded by lateral claddings with 20 nanorods on both sides. Here, the lateral claddings are designed to achieve sufficiently small ${C}_{e,i}$, such that the emission is dominated by surface radiation when the bulk region is sufficiently large. There exist other designs of claddings to achieve the flat fundamental mode, but they may not be able to minimize ${C}_{e,i}$. We provide more details of the design of the claddings in the Supplementary Information Sections 2 and 4.

Modal profiles and modal losses of the resonant modes are then obtained by solving Maxwell’s equations for complex eigenfrequencies using the finite-element method. Figure 4d–f shows the modal profiles of the fundamental and first-order modes (top panel), and the corresponding electric field distributions of the fundamental modes. For the spatially uniform cavity, the fundamental and first-order modes exhibit sinusoidal envelopes as shown in Fig. 4d. The radiation from the fundamental mode is dominated by surface emission as evidenced by the electric field distribution. In stark contrast, by introducing the spatially varying claddings, the modal profile of the fundamental mode becomes flat within the bulk region and quickly decays to zero at the edges, as shown in Fig. 4e, f. However, without symmetry-breaking air voids, or in other words, in the presence of BIC, the bulk region almost does not radiate in the normal direction, as shown in Fig. 4e. Similar issues exist in the flat PCSEL design with linear dispersions due to the presence of BIC, as we discussed earlier^9^. In the existing designs based on linear dispersion from the Dirac point, it is difficult to construct perturbations that enable radiation from BIC while preserving the flat modal profile. In contrast, in our quasi-flat cavity design based on quadratic dispersion, the surface radiation in our quasi-flat cavity can be significantly enhanced (Fig. 4f). It is important to note that for the small cavity with 100 bulk lattices, the radiation from the claddings is still comparable to the surface radiation. As we will show below, the radiation loss from the claddings will decrease as the size of the bulk region increases, and eventually the radiation will be dominated by the surface emission from the bulk region.

**Fig. 5:**
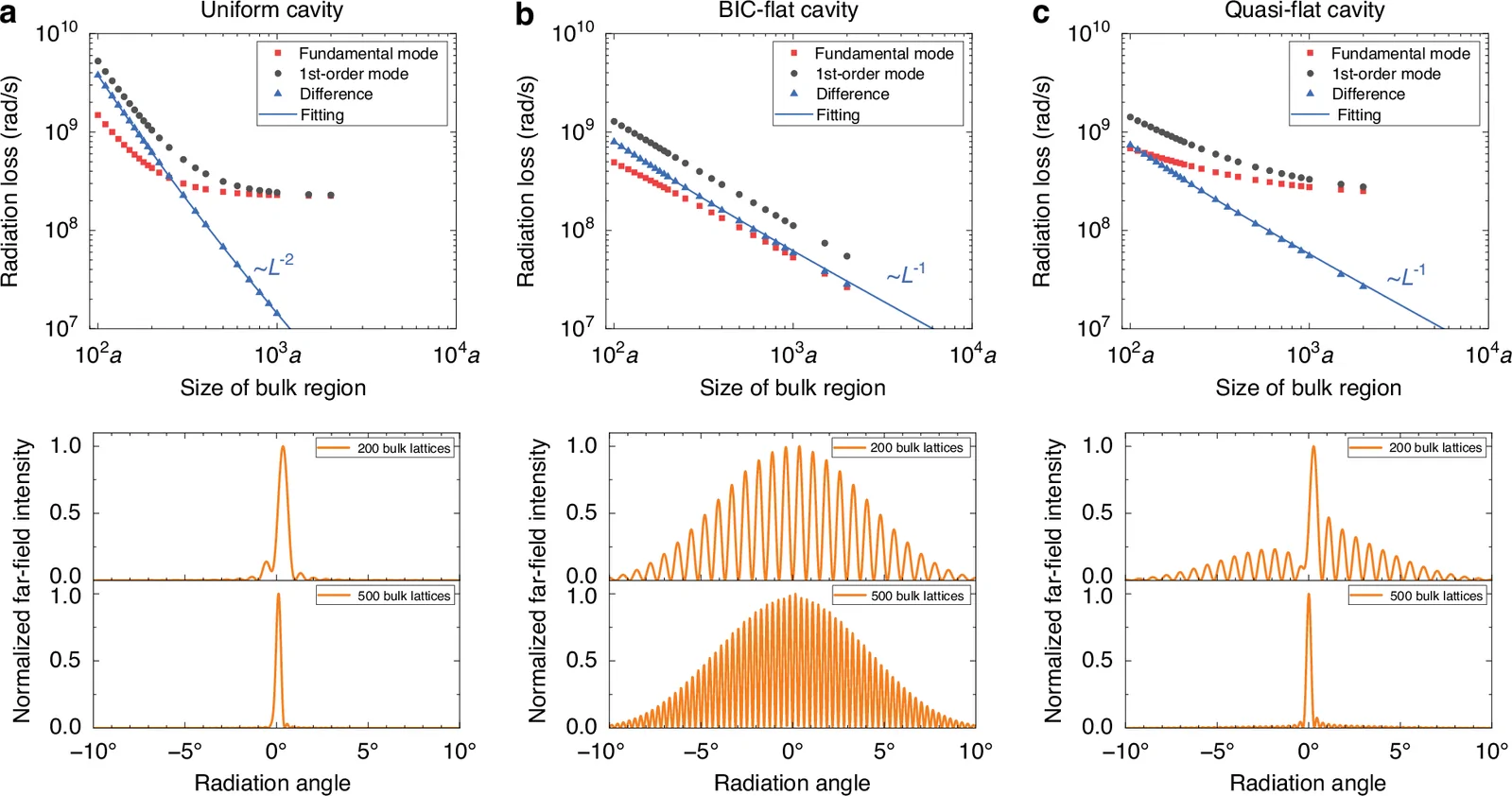
Scaling laws and far-fi eld profi les in 1D finite-size photonic crystals. Scaling of radiation losses (top) and far-field profiles (bottom) in **a** the uniform cavity, **b** the BIC-flat cavity, and **c** our quasi-flat cavity. The uniform cavity exhibits quadratic scaling with the lateral size of the bulk region. Both the BIC-flat cavity and the quasi-flat cavity exhibit linear scaling of radiation losses. The far-field profile of the uniform cavity is always single-lobed, indicating high surface-emission efficiency. The far-field profile of the BIC-flat cavity is always multilobed due to weak surface radiation. In contrast, the far-field profile of our quasi-flat cavity is multilobed for small-scale cavity (200 bulk lattices) but quickly becomes single-lobed as the size of bulk region increases to 500 lattices

We then numerically calculated the scaling of modal losses for all three cavity designs and plotted the results in Fig. 5. The lateral size of the bulk region varies from 100a to 2000a, while the lateral size of the claddings remains the same at 20a. The differential modal loss of the uniform cavity scales quadratically as $\sim {L}^{-2}$ (blue solid line in Fig. 5a). Despite this unfavorable quadratic scaling, the uniform lattice design exhibits an excellent single-lobed far-field pattern for all cavity sizes, indicating high surface-emission efficiency. On the contrary, by incorporating spatially varying truncations on both lateral edges, the differential modal loss scales linearly as $\sim {L}^{-1}$ as we theoretically predicted (Fig. 5b, c), even though the dispersion of the underlying photonic crystal is quadratic. In the presence of BIC, the far-field pattern is always multi-lobed (Fig. 5b) as the radiation is dominated by the radiation from the edges. This issue is resolved by introducing symmetry-breaking perturbations to the unit-cell structures. For small sizes such as 200 bulk lattices, the far-field radiation is multi-lobed but with a dominating peak in the normal direction. As the edge radiation loss decreases with increasing lateral size L and becomes sufficiently small, the far field becomes single-lobed even for a relatively small bulk region, e.g., 500a, as shown in Fig. 5c. It is important to note that the edge radiation of our quasi-flat design can be further reduced by tuning the spatially varying claddings to reduce diffractions.

### Quasi-flat photonic-crystal slab cavity

**Fig. 6:**
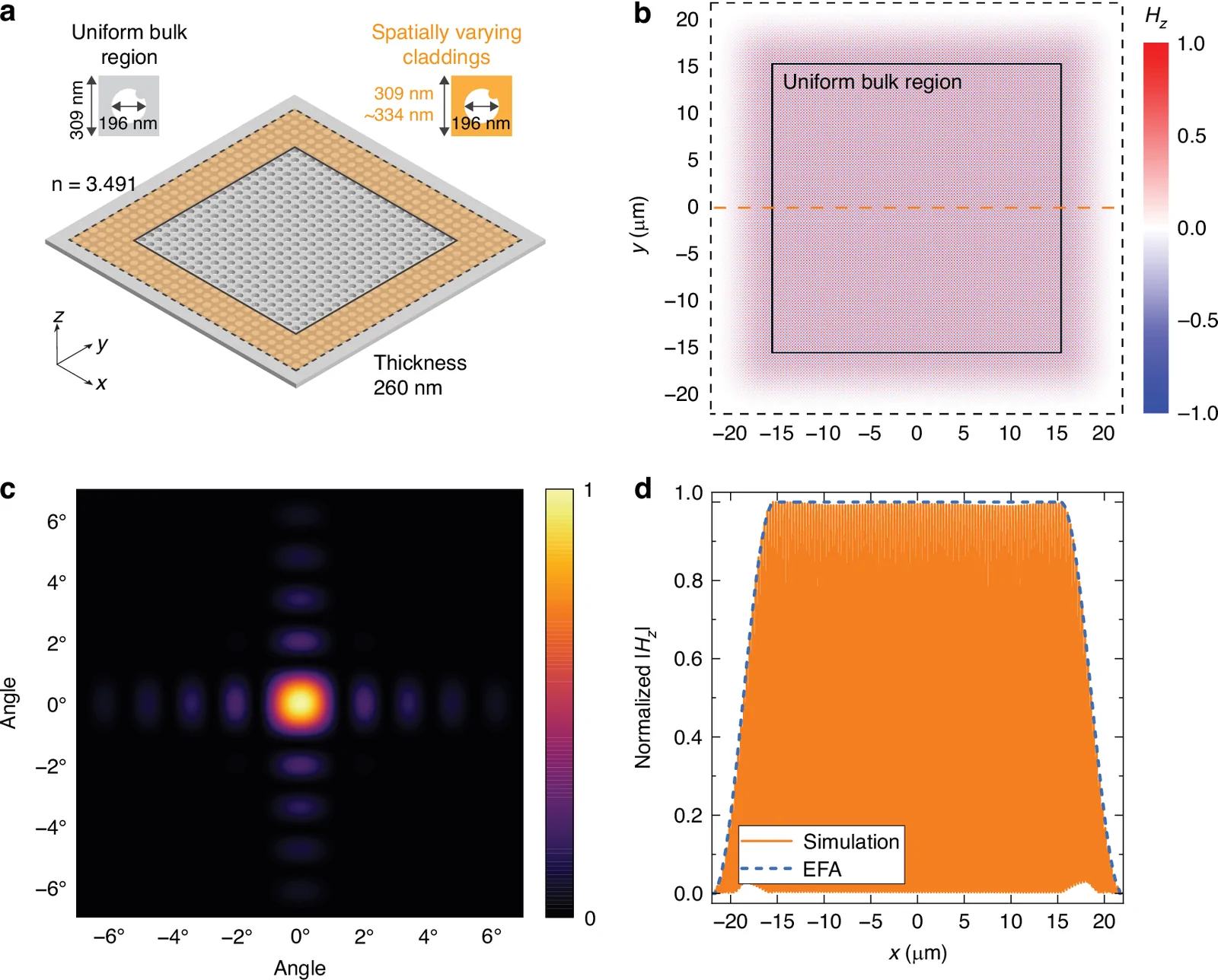
Demonstration of quasi-flat mode in a finite-size photonic-crystal slab. **a** Schematic of the finite-size photonic-crystal slab. It consists of two regions, the bulk region (gray region within the solid rectangle) with spatially uniform lattice constants, and the spatially varying claddings (orange region) with nonuniform lattice constants. The diameter of the airholes is $196{nm}$. **b** Normalized magnetic field distribution ${H}_{z}$ of the fundamental mode with transverse electric polarization. The field envelope is uniform within the uniform bulk region. **c** Far-field intensity profile of the fundamental mode. **d** Normalized magnitude of the magnetic field $\left|{H}_{z}\right|$ along the y=0 line (orange dashed line in **b**). The blue dashed line denotes the envelope as obtained from the EFA

Finally, we numerically demonstrate and validate that our quasi-flat cavity design can be realized in a photonic-crystal slab in 3D space. We consider a dielectric photonic-crystal slab as shown in Fig. 6a. It consists of square lattices of circular airholes in a dielectric slab with a refractive index of 3.491 and a thickness of $260{nm}$. The radius of the airholes is $98{nm}$. A small circular perturbation with a radius of $30{nm}$ is introduced to break the symmetry of the unit cell and increase surface radiation. The photonic crystal consists of two regions, the uniform region with 100 by 100 lattices and a fixed lattice constant of $309{nm}$, and the lateral claddings with 20 lattices and spatially varying lattice constants. The total size of the photonic crystal is 140 by 140 lattices. The corresponding laterally infinite photonic crystal supports a quadratic band edge for transverse electric (TE) polarization. In the bulk region, the corresponding band edge frequency is $336.85{THz}$. In the lateral claddings, the band edge frequency varies with the lattice constants. The details of the spatially varying lattice constants are provided in the Supplementary Information Section 2.

We solve for the resonant modes of the photonic-crystal slab using Tidy3D, a commercially available large-scale finite-difference time-domain (FDTD) solver^34^. Point sources with the temporal profile of a Gaussian pulse are placed inside the slab to excite the resonant modes, and the decay of the electric field inside the slab as a function of time is recorded. We use harmonic inversion^35^ to analyze the recorded time series and obtain the resonant frequencies. We then perform another FDTD simulation to record the lateral field distribution at the resonant frequencies using the technique of discrete-time Fourier transformation^36^. Figure 6b shows the magnetic field ${H}_{z}$ distribution of the fundamental mode, and Fig. 6d shows a line cut of the magnetic field distribution at y=0 (orange dashed line in Fig. 6b). As expected, the field envelope is flat in the bulk region and quickly decays to zero in the claddings, validating our design. Furthermore, we calculate the far-field pattern of the fundamental mode using near-field to far-field transformation and plot the result in Fig. 6c. The far field is clearly single-lobed, even though the size of the bulk region considered here is relatively small (100 by 100 periods or 30.9μm by 30.9μm). The side lobes will be significantly reduced as the size of the bulk region scales up. Since full-wave simulation of photonic-crystal slabs exceeding 200 by 200 periods is beyond the capabilities of computational resources available to us, we provide a numerical example using our EFA formalism to demonstrate the improved far-field radiation profile as the bulk region scales up in area.

### Effect of fabrication imperfections and spatial localization

In the above analysis, we focus on the mode competition between large-area lasing modes and the scaling of the threshold difference between them. However, in practice, the presence of fabrication imperfections can strongly trap the modes in different small-area domains within the cavity, resulting in many spatially non-overlapping small-area single-mode lasers with similar lasing thresholds. The spatial extent of such small-area lasing modes is determined by the localization length ${L}_{{local}}$ associated with the disorders in the cavity. Consequently, to prevent onset of spatially non-overlapping modes, the cavity length L must be within or comparable to the localization length ${L}_{{local}}$. For large-area single-mode lasing, a large localization length ${L}_{{local}}$ is highly desired.

Here we adopt the optimal fluctuation theory^37^ to qualitatively analyze the localization length of PCSEL cavities in the presence of fabrication disorders. Because of the fabrication fluctuations across different unit cells, the band edge frequencies of the unit cells exhibit spatial fluctuations $\triangle \omega \left(x,y\right)$ around the designed frequency ${\omega }_{0}$. The disorder strength is then quantified by the spatial variance ${\sigma }_{{\omega }_{0}}$.

In the framework of optimal fluctuation theory, the random fluctuation forms a random potential well with a domain size of ${L}_{{local}}$ and a potential depth given by $\Delta {\omega }_{{rms}}=\frac{{\sigma }_{{\omega }_{0}}}{{L}_{{local}}}$. Any mode with a kinetic energy lower than the potential depth is trapped within the potential well. In the case of quadratic dispersions, i.e., $\omega \left({\boldsymbol{k}}\right)={\omega }_{{\mathbf{\Gamma }}}+A{\left|{\boldsymbol{k}}\right|}^{2}$, the kinetic energy of the modes scales as $\left|A\right|{L}_{{local}}^{-2}$, and the localization length can be approximated as (see Supplementary Information Section 6 for details)

15 $${L}_{{local}}\approx \frac{{|A|}}{{\sigma }_{{\omega }_{0}}}$$

Therefore, for quadratic band edge, the localization length is proportional to the band curvature $\left|A\right|$ and is often limited due to small $\left|A\right|$, limiting the maximum area for single-mode lasing. In Supplementary Information Section 6, we provide numerical analysis of the effects of disorders on the mode profiles of our quasi-flat cavities.

Similar analysis can also be applied to linear dispersion. In stark contrast, in the case of linear dispersion, the kinetic energy of the modes scales as $\left|{A}_{{linear}}\right|{L}_{{local}}^{-1}$. The modes thus are spatially extended for typical fabrication imperfections with relatively small spatial variance ${\sigma }_{{\omega }_{0}}$ and $\left|{A}_{{linear}}\right|\ge {\sigma }_{{\omega }_{0}}$. Consequently, linear dispersion typically offers much longer localization length than quadratic dispersion^24^.

## Discussion

Because our quasi-flat PCSEL design only relies on engineering the lateral claddings of uniform photonic crystals, the threshold difference can be further enhanced by engineering the unit-cell structure in the bulk region, while maintaining the linear scaling of differential modal loss $\Delta \alpha \sim {L}^{-1}$ and high spatial overlap factor ${\mathcal{F}} \sim 1$. Notable examples include triangular airholes^6^, double-lattice^7,8,11^ and triple-lattice^12^ unit-cell designs. The in-plane feedback introduced by such unit-cell structures can significantly increase the constant ${C}_{s}$ and thus increase the differential modal loss $\Delta \alpha$^8^. On the other hand, while we have only considered spatially varying the lattice constants to engineer the lateral claddings, our concept can be extended to other approaches of engineering the lateral boundaries, such as modification of the unit-cell structure^10,38^ in the claddings, introducing gain or loss around the bulk region^39,40^, and utilization of photonic topological insulators^41,42^. These approaches provide more degrees of freedom to engineer the boundary conditions at the lateral bulk-cladding interfaces. We expect incorporating these approaches into our design to further increase the threshold difference by increasing the constant ${C}_{e,i}$, or even modifying the power exponent l of edge-loss difference $\Delta {\alpha }_{e} \sim {L}^{-l}$. On the other hand, the spatial profile of gain distribution, i.e., $G({\boldsymbol{r}})$ in Eq. (10), can also be optimized to improve the modal overlap.

Besides optimizing threshold difference and mode competition, extending the localization length is also critical to mitigate the effects of spatial localization due to fabrication disorders. In the case of quadratic dispersion, while it is possible to increase the band curvature and the localization length by optimizing the unit cell structure, the enhancement may not be sufficient to extend the localization length to millimeter or even centimeter scale. Linear dispersion is fundamentally favorable over quadratic dispersion for achieving long localization lengths, but it also exhibits the tradeoff of low surface-emission efficiency. Therefore, more complex band structures and PCSEL designs that retain the favorable properties for large-area single-mode lasing while mitigating the tradeoffs are required to address this challenge.

We emphasize that we consider a single isolated quadratic band in our design as a proof of concept. In more practical designs of electrically pumped PCSELs, there typically exist multiple bands with both quadratic and linear dispersion terms. Our concept can be extended to these structures, but a more involved model that considers the effects of multiple bands is required. Furthermore, effects such as carrier injection, photon-carrier interaction and thermal effects can change the refractive index of the photonic crystal and thus shift the band edge frequencies. Therefore, it is necessary to consider such effects in the modeling and design of photonic-crystal slab cavities. Because of the large scale of PCSELs, efficient numerical solvers of Maxwell–Bloch equations and optimization algorithms will be required to scale up our theoretical proposal to ultra-large-area ultra-high-power single-mode PCSELs.

In conclusion, we have developed a theoretical framework to elucidate the interplay between the physics of mode competition and the modal properties in photonic-crystal-slab surface-emitting lasers. In general, the goal of a scalable PCSEL design is to sustain an as-large-as-possible threshold difference between the fundamental and higher-order modes as the lasing emission area increases, such that ultra-high-power single-mode lasing can be realized even at ultra-large emission area. Using our theoretical framework, we provide the conditions required to realize this goal, which include favorable scaling of differential modal loss such as $\triangle \alpha \sim {L}^{-1}$, significant spatial overlap with ${\mathcal{F}}{\mathscr{\to }}1$, and controllable radiation dispersion ${\gamma }_{s}({\boldsymbol{k}})$ and edge radiation loss. Existing scalable PCSEL designs typically do not satisfy all conditions, leaving significant room for further improvement. We provide an alternative scalable PCSEL design that satisfies all conditions and numerically validate our design using full-wave simulations. Our results thus open new pathways towards single-mode PCSEL. Moreover, the physics and concepts developed in our work can be potentially applied to other laser cavities such as VCSEL and distributed feedback lasers, and engineering of other beam characteristics such as polarization states^43,44^ and spatial profiles^45–47^, providing new possibilities for laser cavity design.

## Materials and methods

The 2D simulations in Fig. 4 and Fig. 5 are obtained from the commercial software COMSOL using eigenfrequency solutions. The 3D simulation Fig. 6 is obtained from the commercial software Tidy3D.

## Supplementary information

Supplementary Information

## Data Availability

The data that support the findings of this study may be available from the first author and the corresponding author on reasonable request.
