# Supplementary material for: General photonic-crystal slab cavity design for scalable high-power single-mode lasing
Source: Light Sci Appl. 2026 Aug 20;15:353. doi: 10.1038/s41377-026-02448-6 (PMC13493973; doi:10.1038/s41377-026-02448-6)
Supplement: Supplementary file 1 [file 41377_2026_2448_MOESM1_ESM.pdf]

Supplementary Information for

General photonic-crystal slab cavity design for scalable high-power single-mode lasing

Ming Zhou,[1] Mingsen Pan,[2] Chhabindra Gautam,[2] Subhashree Seth,[3] Thomas Rotter,[3] Ganesh Balakrishnan,[3] Weidong Zhou,[2] Shanhui Fan[1,*]

1. *Department of Electrical Engineering, Stanford University, Stanford, California 94305, USA*
2. *Department of Electrical Engineering, University of Texas at Arlington, Arlington, TX 76019, USA*
3. *Department of Electrical and Computer Engineering, University of New Mexico, Albuquerque, New Mexico 87131, USA*

* Correspondence to: Shanhui Fan (shanhui@stanford.edu)

1. Derivation of the envelope function equations

Here we provide detailed discussions of our theoretical formalism. Without losing generality, we consider a finite-size photonic-crystal slab embedded in air. The whole structure thus can be divided into two regions: the photonic-crystal slab and the surrounding air. We will first treat each region separately as infinite continuum with given band dispersion. We carry out multi-scale analysis and derive the equation of envelope function for each region. We then treat the finite-size effect by applying proper boundary conditions to the envelope functions at the interface between the two regions.

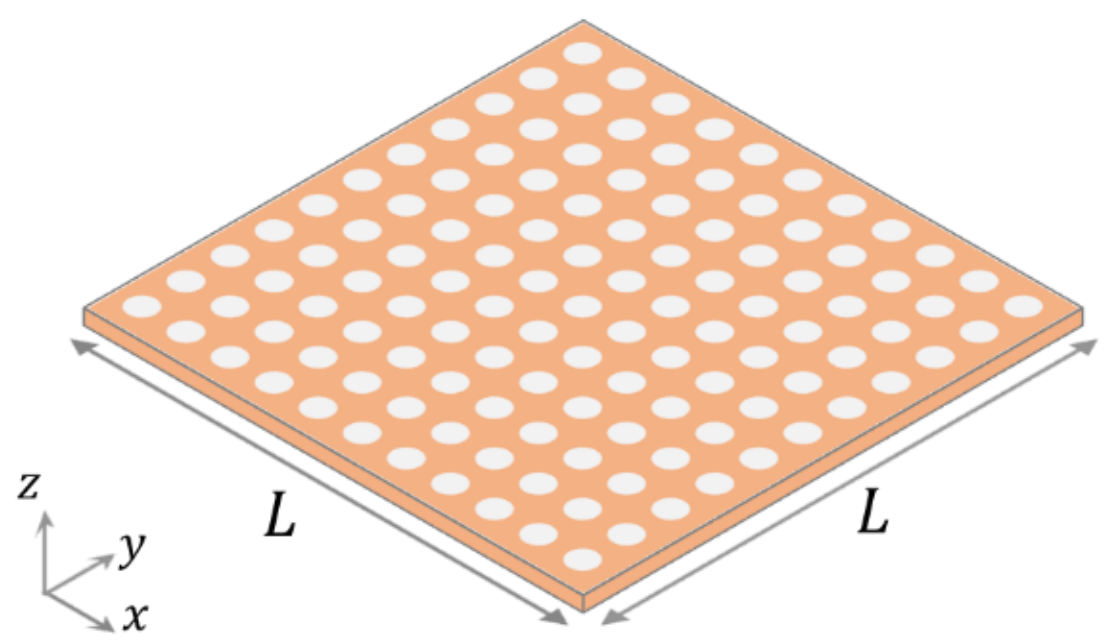

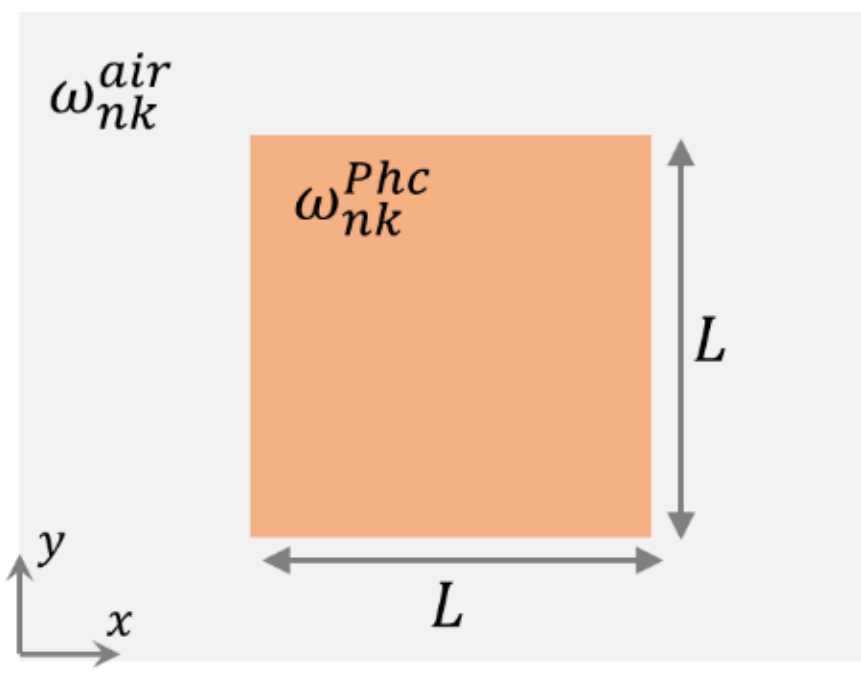

Fig. S1. Schematic of a finite-size photonic-crystal slab in air. The lateral size of the slab is L in both x and y directions. We separate the whole structure into two regions: the photonic-crystal region and the surrounding air. Each region is then treated as an infinite continuum with given band dispersion ω_{nk}.

We start with the photonic-crystal-slab region, in which the modes must satisfy the following wave equation

$$\nabla \times (\nabla \times \boldsymbol{E}(\boldsymbol{r})) = \frac{\Omega^2}{c^2} \epsilon_0(\boldsymbol{r}) \boldsymbol{E}(\boldsymbol{r}) \tag{S1}$$

where $\boldsymbol{E}(\boldsymbol{r})$ and Ω are the electric field and the complex eigenfrequency of the resonant mode in the photonic-crystal slab, respectively. c is the vacuum speed of light. Here, we assume the dielectric function $\epsilon_0(\boldsymbol{r})$ is infinitely periodic along the lateral x and y directions. Throughout our derivation, we use Ω and ω to differentiate complex frequency and real frequency, and the imaginary part of Ω describes the radiative decay rate of the resonant mode.

In general, we expect that the field distribution in a finite-size photonic crystal slab is a product of a slowly varying envelope function with the modal function of the infinite periodic photonic crystal. Our goal is to derive from Eq. (S1), which describes the property of infinite periodic photonic crystal, an equation that governs the behavior of the envelope function that can be used to describe the finite-size crystal.

For this purpose, we apply the multiscale method to the lateral x and y directions by introducing different length scales, $x_\alpha = \mu^\alpha x$ and $y_\alpha = \mu^\alpha y$, where $\mu \ll 1$ and $\alpha = 0,1,2, \dots$. We denote $\boldsymbol{r_0} = (x_0, y_0, z)$ and $\boldsymbol{r_\alpha} = (x_\alpha, y_\alpha)$ for $\alpha \geq 1$. The spatial derivatives along the lateral directions then can be written as

$$\frac{\partial}{\partial x}=\frac{\partial}{\partial x_0}+\mu\frac{\partial}{\partial x_1}+\mu^2\frac{\partial}{\partial x_2}+\cdots \tag{S2}$$

$$\frac{\partial}{\partial y}=\frac{\partial}{\partial y_0}+\mu\frac{\partial}{\partial y_1}+\mu^2\frac{\partial}{\partial y_2}+\cdots \tag{S3}$$

Therefore, the gradient operator can be written as:

$$\nabla=\nabla_0+\mu\nabla_1+\mu^2\nabla_2+\cdots \tag{S4}$$

where

$$\nabla_0=\hat{\boldsymbol{x}}\frac{\partial}{\partial x_0}+\hat{\boldsymbol{y}}\frac{\partial}{\partial y_0}+\hat{\boldsymbol{z}}\frac{\partial}{\partial z}$$

$$\nabla_1=\hat{\boldsymbol{x}}\frac{\partial}{\partial x_1}+\hat{\boldsymbol{y}}\frac{\partial}{\partial y_1}$$

$$\nabla_2=\hat{\boldsymbol{x}}\frac{\partial}{\partial x_2}+\hat{\boldsymbol{y}}\frac{\partial}{\partial y_2}$$

Similarly, the field $\boldsymbol{E}(\boldsymbol{r})$ can be expanded as[1]

$$\boldsymbol{E}(\boldsymbol{r})=\mu\boldsymbol{e_1}+\mu^2\boldsymbol{e_2}+\mu^3\boldsymbol{e_3}+\cdots \tag{S5}$$

And the complex eigenfrequency Ω can be expanded as:

$$\Omega=\Omega_0+\mu\Delta\Omega_1+\mu^2\Delta\Omega_2+\cdots \tag{S6}$$

Below, we insert Eqs. (S4-S6) into Eq. (S1) and consider the expansion in the increasing order of μ. In this expansion, the dielectric function is taken as $\epsilon_0(\boldsymbol{r}_0)$ since the structure we are considering here is an infinite periodic photonic crystal and therefore varies only at the scale of r_0.

Bloch modes. The Bloch modes of the crystal are described by:

$$\nabla_0\times\left(\nabla_0\times\boldsymbol{E}_{n\boldsymbol{k}}(\boldsymbol{r_0})\right)=\frac{\Omega_{nk}^2}{c^2}\epsilon_0(\boldsymbol{r}_0)\boldsymbol{E}_{n\boldsymbol{k}}(\boldsymbol{r_0}) \tag{S7}$$

$\boldsymbol{E}_{n\boldsymbol{k}}(\boldsymbol{r_0})$ and $\Omega_{n\boldsymbol{k}}$ are the Bloch mode and the complex frequency associated with the nth band at wave vector $\boldsymbol{k}$, respectively. The Bloch modes satisfy the orthonormal equation:

$$\int dV_0\,\epsilon_0(\boldsymbol{r}_0)\boldsymbol{E}_{m\boldsymbol{k}}^*(\boldsymbol{r_0})\boldsymbol{E}_{n\boldsymbol{k}}(\boldsymbol{r_0})=\delta_{mn} \tag{S8}$$

Here the integral is performed over the unit cell volume V_0. We note that the Bloch modes take the form

$$\boldsymbol{E}_{n\boldsymbol{k}}(\boldsymbol{r_0})=e^{j\boldsymbol{k}\cdot\boldsymbol{r}_0}\boldsymbol{u}_{n\boldsymbol{k}}(\boldsymbol{r_0}) \tag{S9}$$

where $\boldsymbol{u}_{n\boldsymbol{k}}(r_0)$ denote the periodic part of the Bloch mode. Using $\Omega_0 = \Omega_{nk}$, and Eq. (S6), we have

$$\Omega^2 = \Omega_{n\boldsymbol{k}}^2 + 2\mu\Omega_{n\boldsymbol{k}}\Delta\Omega_1 + \mu^2(\Delta\Omega_1^2 + 2\Omega_{n\boldsymbol{k}}\Delta\Omega_2) + \cdots \tag{S10}$$

This equation will be used in the derivations below.

First-order equation. We collect all terms proportional to μ and obtain

$$\nabla_0 \times (\nabla_0 \times \boldsymbol{e_1}) = \frac{\Omega_{n\boldsymbol{k}}^2}{c^2}\epsilon_0 \boldsymbol{e_1} \tag{S11}$$

This equation involves only the derivative in the smallest scales of $\boldsymbol{r_0}$. Therefore, we can write $\boldsymbol{e_1}$ as

$$\boldsymbol{e_1} = f(\boldsymbol{r_1}, \boldsymbol{r_2}, \dots)\boldsymbol{E}_{nk}(\boldsymbol{r_0}) \tag{S12}$$

where $f(\boldsymbol{r_1}, \boldsymbol{r_2}, \dots)$ is the envelope function that modulates the Bloch modes $\boldsymbol{E}_{nk}(\boldsymbol{r_0})$. Thus, the solution to the first-order equation indeed has the form of a product of an envelope function with the Bloch modes. To obtain the solutions of the envelope function f, we will need to further proceed to higher-order terms of μ.

Second-order equation. We collect the terms proportional to μ^2 and obtain

$$\nabla_0 \times (\nabla_0 \times \boldsymbol{e_2}) - \frac{\Omega_{n\boldsymbol{k}}^2}{c^2}\epsilon_0 \boldsymbol{e_2} = \frac{2\Omega_{n\boldsymbol{k}}\Delta\Omega_1}{c^2}\epsilon_0 \boldsymbol{e_1} - \nabla_0 \times (\nabla_1 \times \boldsymbol{e_1}) - \nabla_1 \times (\nabla_0 \times \boldsymbol{e_1}) \tag{S13}$$

To solve this equation, we can also expand the field $\boldsymbol{e_2}$ in terms of the Bloch modes as [1]

$$\boldsymbol{e_2} = \sum_{m \neq n} g_{m\boldsymbol{k}}(\boldsymbol{r_1}, \boldsymbol{r_2}, \dots)\boldsymbol{E}_{mk}(\boldsymbol{r_0}) \tag{S14}$$

where $g_{m\boldsymbol{k}}$ is a new set of envelope functions that are relevant at this order and the band index m goes over all bands except the nth band. Substituting Eq. (S12) and (S14) into Eq. (S13), we obtain

$$\begin{aligned} &\sum_{m \neq n} \left(\frac{\Omega_{m\boldsymbol{k}}^2}{c^2} - \frac{\Omega_{n\boldsymbol{k}}^2}{c^2}\right) g_{m\boldsymbol{k}}\, \epsilon_0 \boldsymbol{E}_{mk} \\ &= \frac{2\Omega_{nk}\Delta\Omega_1}{c^2}\epsilon_0 f\boldsymbol{E}_{nk} - \nabla_0 \times (\nabla_1 \times f\boldsymbol{E}_{nk}) - \nabla_1 \times (\nabla_0 \times f\boldsymbol{E}_{nk}) \end{aligned} \tag{S15}$$

Using the form of the Bloch mode in Eq. (S9), we can simplify the 2nd and 3rd term on the right-hand side of Eq. (S15) as

$$\nabla_0 \times (\nabla_1 \times f\boldsymbol{E}_{nk}) = e^{j\boldsymbol{k}\cdot\boldsymbol{r_0}}(j\boldsymbol{k} + \nabla_0) \times [\nabla_1 f \times \boldsymbol{u}_{n\boldsymbol{k}}] \tag{S16}$$

$$\nabla_1 \times (\nabla_0 \times f\boldsymbol{E}_{nk}) = e^{j\boldsymbol{k}\cdot\boldsymbol{r_0}}\nabla_1 f \times [(j\boldsymbol{k} + \nabla_0) \times \boldsymbol{u}_{n\boldsymbol{k}}] \tag{S17}$$

Here we have used the identity that $\nabla \times (\varphi \boldsymbol{U}) = \varphi \nabla \times \boldsymbol{U} + \nabla \varphi \times \boldsymbol{U}$. We then multiply both sides of Eq. (S15) by $\boldsymbol{E}_{n\boldsymbol{k}}^*$ and integrate over a unit cell. Combining with Eqs. (S16-S17) and the orthogonality of Bloch modes in Eq. (S8), we obtain

$$\frac{2\Omega_{n\boldsymbol{k}}\Delta\Omega_1}{c^2} f - \langle \boldsymbol{u}_{n\boldsymbol{k}} | \nabla_1 f \cdot (\nabla_{\boldsymbol{k}} \boldsymbol{\mathcal{H}}_{\boldsymbol{k}}) | \boldsymbol{u}_{n\boldsymbol{k}} \rangle = 0 \tag{S18}$$

The derivative $\nabla_{\boldsymbol{k}}$ is defined as $\nabla_{\boldsymbol{k}} = \frac{\partial}{\partial k_x}\hat{\boldsymbol{x}} + \frac{\partial}{\partial k_y}\hat{\boldsymbol{y}}$**.** We adopt the notation of Dirac brackets for simplicity. The operator $\boldsymbol{\mathcal{H}}_{\boldsymbol{k}}$ is defined as [2]

$$\boldsymbol{\mathcal{H}}_{\boldsymbol{k}} | \boldsymbol{u}_{n\boldsymbol{k}} \rangle = (\nabla_0 + j\boldsymbol{k}) \times [(\nabla_0 + j\boldsymbol{k}) \times | \boldsymbol{u}_{n\boldsymbol{k}} \rangle] \tag{S19}$$

In deriving the above equations, we have also used the fact that $\left\langle \boldsymbol{u}_{n\boldsymbol{k}} \middle| \frac{\partial}{\partial k_i} \boldsymbol{u}_{n\boldsymbol{k}} \right\rangle = 0$, where $i = x, y$ [2].

By further invoking the relation $\langle \boldsymbol{u}_{n\boldsymbol{k}} | (\nabla_{\boldsymbol{k}} \boldsymbol{\mathcal{H}}_{\boldsymbol{k}}) | \boldsymbol{u}_{n\boldsymbol{k}} \rangle = -\frac{1}{c^2} \nabla_{\boldsymbol{k}} \omega_{n\boldsymbol{k}}^2$ around the wave vector $\boldsymbol{k}$, where $\omega_{n\boldsymbol{k}}$ is the real part of the complex frequency $\Omega_{n\boldsymbol{k}}$, we obtain that

$$\nabla_{\boldsymbol{k}} \omega_{n\boldsymbol{k}}^2 \cdot \nabla_1 f + 2\Omega_{n\boldsymbol{k}} \Delta\Omega_1 f = 0 \tag{S20}$$

Since we are considering the band edge at $\boldsymbol{\Gamma}$, i.e., $\boldsymbol{k} = \boldsymbol{0}$, both $\frac{\partial \omega_{n\boldsymbol{k}}^2}{\partial k_x} = 0$ and $\frac{\partial \omega_{n\boldsymbol{k}}^2}{\partial k_y} = 0$. Therefore, we must have $\Delta\Omega_1 = 0$ such that Eq. (S20) holds true for any envelope function f. In such cases, equation (S20) is always satisfied, and we will need to proceed to higher-order terms of μ to obtain the solution for f.

Third-order equation. We further consider terms with μ^3, which leads to

$$\begin{aligned} &\nabla_0 \times (\nabla_0 \times \boldsymbol{e_3}) - \frac{\Omega_{n\boldsymbol{k}}^2}{c^2} \epsilon_0 \boldsymbol{e_3} \\ &= \frac{2\Omega_{nk}\Delta\Omega_1}{c^2} \epsilon_0 \boldsymbol{e_2} - \nabla_0 \times (\nabla_1 \times \boldsymbol{e_2}) - \nabla_1 \times (\nabla_0 \times \boldsymbol{e_2}) \\ &+ \frac{\Delta\Omega_1^2 + 2\Omega_{n\boldsymbol{k}}\Delta\Omega}{c^2} \epsilon_0 \boldsymbol{e_1} - \nabla_0 \times (\nabla_2 \times \boldsymbol{e_1}) - \nabla_2 \times (\nabla_0 \times \boldsymbol{e_1}) - \nabla_1 \times (\nabla_1 \times \boldsymbol{e_1}) \end{aligned} \tag{S21}$$

Since we are specifically looking for envelope functions f that only vary on the scales $\boldsymbol{r_1}$, i.e., $\nabla_1 f \neq 0$ and $\nabla_2 f = 0$, we can ignore terms contain ∇_2. Multiplying both sides of Eq. (S21) with $\boldsymbol{E}_{n\boldsymbol{k}}^*$ and integrating over a unit cell, and further substituting the expressions of $\boldsymbol{e_1}$, $\boldsymbol{e_2}$ and $\boldsymbol{E}_{n\boldsymbol{k}}$, we obtain

$$\sum_{m \neq n} \langle \boldsymbol{u}_{n\boldsymbol{k}} | \nabla_1 g_{m\boldsymbol{k}} \cdot (\nabla_{\boldsymbol{k}} \boldsymbol{\mathcal{H}}_{\boldsymbol{k}}) | \boldsymbol{u}_{m\boldsymbol{k}} \rangle + \langle \boldsymbol{u}_{n\boldsymbol{k}} | \nabla_1 \times (\nabla_1 f \times | \boldsymbol{u}_{n\boldsymbol{k}} \rangle) = \frac{\Delta\Omega_1^2 + 2\Omega_{n\boldsymbol{k}}\Delta\Omega_2}{c^2} f \tag{S22}$$

In order to eliminate $g_{m\boldsymbol{k}}$ in Eq. (S22), we multiply both sides of Eq. (S15) with $\boldsymbol{E}_{m\boldsymbol{k}}^*$ and integrate over a unit cell to obtain

$$g_{m\boldsymbol{k}} = \frac{c^2}{\omega_{n\boldsymbol{k}}^2 - \omega_{m\boldsymbol{k}}^2} \langle \boldsymbol{u}_{m\boldsymbol{k}} | \nabla_1 f \cdot (\nabla_k \boldsymbol{\mathcal{H}}_{\boldsymbol{k}}) | \boldsymbol{u}_{n\boldsymbol{k}} \rangle \tag{S23}$$

Here we ignore the imaginary parts of $\Omega_{n\boldsymbol{k}}$ and $\Omega_{m\boldsymbol{k}}$, because they are typically much smaller than the corresponding real parts for resonant modes in photonic-crystal slab around $\boldsymbol{k} = \boldsymbol{0}$. On the other hand, the second term on the left-hand side of Eq. (S22) can be written as

$$\langle \boldsymbol{u}_{n\boldsymbol{k}} | \nabla_1 \times (\nabla_1 f \times | \boldsymbol{u}_{n\boldsymbol{k}} \rangle) = -\langle \boldsymbol{u}_{n\boldsymbol{k}} | \nabla_1 \cdot (\nabla_{\boldsymbol{k}} \otimes \nabla_{\boldsymbol{k}} \boldsymbol{\mathcal{H}}_{\boldsymbol{k}}) \cdot \nabla_1 f | \boldsymbol{u}_{n\boldsymbol{k}} \rangle \tag{S24}$$

where $\boldsymbol{U} \otimes \boldsymbol{V}$ denotes the tensor product between two vectors $\boldsymbol{U}$ and $\boldsymbol{V}$. We then substitute Eq. (S23-S24) back into Eq. (S22). The resulting equation takes the form

$$\nabla_1 \cdot \boldsymbol{A} \cdot \nabla_1 f = \frac{\Delta\Omega_1^2 + 2\Omega_{nk}\Delta\Omega_2}{c^2} f \tag{S25}$$

where $\boldsymbol{A}$ is a tensor with its element A_{ab} defined as

$$A_{ab} = -\frac{1}{2} \left\langle \boldsymbol{u}_{nk} \middle| \frac{\partial^2 \boldsymbol{\mathcal{H}}_{\boldsymbol{k}}}{\partial k_a \partial k_b} \middle| \boldsymbol{u}_{nk} \right\rangle + \sum_{m \neq n} \frac{c^2 \left\langle \boldsymbol{u}_{mk} \middle| \frac{\partial \boldsymbol{\mathcal{H}}_{\boldsymbol{k}}}{\partial k_a} \middle| \boldsymbol{u}_{nk} \right\rangle \left\langle \boldsymbol{u}_{nk} \middle| \frac{\partial \boldsymbol{\mathcal{H}}_{\boldsymbol{k}}}{\partial k_b} \middle| \boldsymbol{u}_{mk} \right\rangle}{\omega_{n\boldsymbol{k}}^2 - \omega_{m\boldsymbol{k}}^2} \tag{S26}$$

where $a, b = x, y$. By further applying perturbation theory, we find that [2] $A_{ab} = -\frac{1}{2c^2} \frac{\partial^2 \omega_{n\boldsymbol{k}}^2}{\partial k_a \partial k_b} |_{\boldsymbol{k}=\boldsymbol{0}}$. Thus, we can rewrite (Eq. S25) as

$$\frac{1}{2} \nabla_1 \cdot \boldsymbol{W}^{(PhC)} \cdot \nabla_1 f + 2\Omega_{n\boldsymbol{k}} (\Omega - \Omega_{n\boldsymbol{k}}) f = 0 \tag{S27}$$

Here $\boldsymbol{W}^{(PhC)}$ is an order-2 tensor with its element defined as $W_{ab}^{(PhC)} = \frac{\partial^2 \omega_{n\boldsymbol{k}}^2}{\partial k_a \partial k_b} |_{\boldsymbol{k}=\boldsymbol{0}}$, where $a, b = x, y$. It is also important to recall that we are considering the bandgap edge at $\boldsymbol{\Gamma}$, thus $\Delta\Omega_1 = 0$. This equation is sufficient for solving the envelope function f and we will truncate our expansion here.

Envelope function equation for air. We can apply the same procedures described above to the surrounding air. For air, since the system is sufficiently simple, we can choose as the starting point the Bloch mode at $\boldsymbol{\Gamma}$ with the frequency $\omega_{n\boldsymbol{\Gamma},air} = 0$ and with $\frac{\partial \omega_{n\boldsymbol{\Gamma}.air}^2}{\partial k_x} = 0$. The frequency correction term appears in the envelope function equation for air is $\Delta\Omega_{1,air}^2$ Therefore, the envelope function for the Bloch mode at $\boldsymbol{\Gamma}$ in the surrounding air can be written as

$$\frac{1}{2} \nabla_1 \cdot \boldsymbol{W}^{(air)} \cdot \nabla_1 f + \Omega^2 f = 0 \tag{S28}$$

Here we use the fact that $\Delta\Omega_{1,air} = \Omega$. $\boldsymbol{W}^{(air)}$ is a tensor with its element defined as $W_{ab}^{(air)} = \frac{\partial^2 c^2 |\boldsymbol{k}|^2}{\partial k_a \partial k_b} |_{\boldsymbol{k}=\boldsymbol{0}}$.

In general, Eq. (S27-28) should be solved together with proper boundary conditions at the photonic-crystal-air interface. For the specific structure considered in the main text, where the field envelope at the interface is minimized, solving Eq. S27 is sufficient as we will show below.

2. Design procedure of quasi-flat cavities with spatially varying claddings

To design a cavity with a quasi-flat fundamental mode, we start with the desired envelope function $f_d(x,y)$. As we discussed in the main text, a quasi-flat cavity consists of a bulk region with lateral dimensions of L, and spatially varying claddings with a thickness of L_c. To facilitate the EFA formulism, the desired envelope function $f_d(x,y)$ and its derivative should be continuous at the perimeter of the bulk region, i.e., $|x| = \frac{L}{2}$ and $|y| = \frac{L}{2}$. Meanwhile, it should decay to zero at the edges $|x| = \frac{L}{2} + L_c$ and $|y| = \frac{L}{2} + L_c$. One potential choice that satisfies these conditions is the raised cosine function. The corresponding $f_d(x,y)$ then is given by

$$f_d(x,y) = \begin{cases} 1, |x| \le \frac{L}{2}, |y| \le \frac{L}{2} \\ \frac{1}{2} + \frac{1}{2}\cos\frac{\pi}{L_c}\left(|x| - \frac{L}{2}\right), \frac{L}{2} < |x| \le \frac{L}{2} + L_c \\ \frac{1}{2} + \frac{1}{2}\cos\frac{\pi}{L_c}\left(|y| - \frac{L}{2}\right), \frac{L}{2} < |y| \le \frac{L}{2} + L_c \end{cases} \quad \text{(S29)}$$

It is important to note that there exist other choices of envelope functions that satisfy the continuity requirements, e.g., sigmoid functions. We consider the above raised cosine function as a particular example as it clearly defines a bulk-cladding interface.

For spatially nonuniform structures, we can assume each unit cell is locally periodic but with different complex frequency $\Omega_{n\boldsymbol{k}}(x,y)$. If the spatial variation of is relatively small and smooth, the corresponding envelope equation can be readily written as

$$\frac{1}{2}\nabla_1 \cdot \boldsymbol{W}^{(PhC)} \cdot \nabla_1 f + 2\Omega_{n\boldsymbol{k}}(x)\big(\Omega - \Omega_{n\boldsymbol{k}}(x)\big)f = 0 \quad \text{(S30)}$$

We can then substitute the desired envelope function $f_d(x,y)$ into Eq. (S30). Note the eigenfrequency of the fundamental mode is close to the complex frequency Ω_0 associated with the bulk region. Therefore, the envelope function equation becomes

$$\frac{1}{2}\nabla_1 \cdot \boldsymbol{W}^{(PhC)} \cdot \nabla_1 f_d + 2\Omega_{n\boldsymbol{k}}(x)\big(\Omega_0 - \Omega_{n\boldsymbol{k}}(x)\big)f_d \cong 0 \quad \text{(S31)}$$

In the case of isotropic quadratic band edge with $\omega_{n\boldsymbol{k}} = \omega_0 + \frac{k_x^2+k_y^2}{2m_{eff}}$, $W_{ab}^{(PhC)} = \frac{\partial^2 \omega_{n\boldsymbol{k}}^2}{\partial k_a \partial k_b}|_{\boldsymbol{k}=\boldsymbol{0}} = \frac{2\omega_0}{m_{eff}}\delta_{ab}$, where $a, b = x, y$. On the other hand, high-Q quadratic band edges are typically considered for the design of PCSEL. Therefore, we can ignore the imaginary part of the complex frequency Ω and simplify Eq. (S31) to

$$\frac{1}{2m_{eff}}\left(\frac{\partial^2}{\partial x^2}+\frac{\partial^2}{\partial y^2}\right)f_d+\big(\omega_0-\omega(x,y)\big)f_d\cong 0 \qquad \text{(S32)}$$

where $\omega(x,y)$ the spatially varying band-edge frequency at $\mathbf{\Gamma}$ point due to the varying lattice constant. By solving Eq. S32 for $\omega(x,y)$, we obtain the spatially varying band-edge frequency required for a quasi-flat fundamental mode as

$$\omega(x,y)=\omega_0+\frac{1}{2m_{eff}}\frac{f_{d,xx}+f_{d,yy}}{f_d} \qquad \text{(S33)}$$

Here we define $f_{d,xx}=\frac{\partial^2 f_d}{\partial x^2}$ and $f_{d,yy}=\frac{\partial^2 f_d}{\partial y^2}$ for compactness. It is important to note that the above procedure is an approximation as we assume the eigenfrequency of the fundamental mode is the same as the band-edge frequency in the bulk region. Because of this assumption, the resulted envelope profile from the lattice constant profile calculated using Eq. S33 is not exactly flat. For small cavity sizes, we apply a correction factor $\delta<1$ to Eq. S33, which leads to

$$\omega'(x,y)=\omega_0+\frac{(1+\delta)}{2m_{eff}}\frac{f_{d,xx}+f_{d,yy}}{f_d} \qquad \text{(S34)}$$

By fine tuning the correction factor δ, the envelope profile can be made flat in the bulk region.

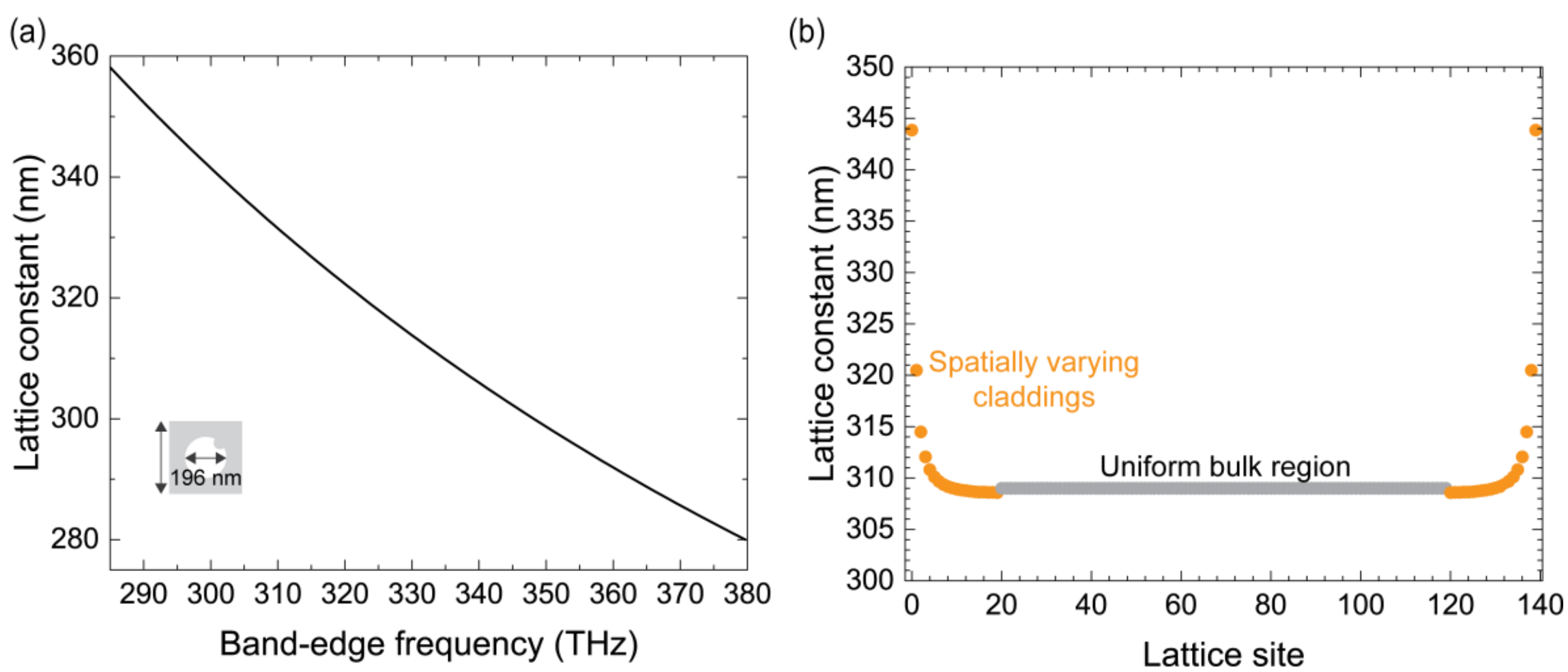

Figure S2. (a) Lattice constant as a function of band-edge frequency. The inset shows the unit-cell structure, which is a square lattice of circular air hole. (b) Spatially varying lattice constant for the structure in Fig. 5.

Next, to find the required spatially varying lattice constants, we need a look-up table that defines the lattice constant as a function of band-edge frequency. Such table can be obtained by sweeping the lattice constant in full-wave simulations and recording the resulting band-edge frequencies. An example table for the lattice constant is shown in Fig. S2a. Here we consider the same photonic crystal demonstrated in Fig. 6, i.e., square lattices of circular airholes in a dielectric slab. Figure S2b shows the lattice constant profile used

in Fig. 6. The lattice-constant profile along both x and y direction is the same, and thus we are only showing the profile along one direction.

It is important to note that there exists a fundamental tradeoff between the edge radiation loss and the length of the cladding region. In general, lower edge radiation loss from the claddings requires longer cladding region. However, as the number of unit cells increases in the cladding region, the relative change between adjacent unit cells becomes smaller, increasing fabrication difficulties. Based on our theoretical calculations, we found that 10-20 periods in the cladding region are sufficient to achieve the desired reflection phase while maintaining relatively low edge radiation losses.

3. Derivation of the gain thresholds for two-mode lasing

The equations of two-mode lasing are given by

$$\frac{d}{dt}I_0 = 2I_0(g - \alpha_0 - \chi_{00}I_0 - \chi_{01}I_1) \tag{S35}$$

$$\frac{d}{dt}I_1 = 2I_1(g - \alpha_1 - \chi_{10}I_0 - \chi_{11}I_1) \tag{S36}$$

$$\chi_{ij} = \int d\boldsymbol{r}\, \varphi_i^2(\boldsymbol{r})\left|\varphi_j^2(\boldsymbol{r})\right|^2 \tag{S37}$$

where I_i and $\varphi_i(\boldsymbol{r})$ are the output intensity and normalized mode profile of the ith mode, respectively. The interaction coefficient χ_{ij} defines the interaction between the ith and j^{th} mode, and the integration in Eq. (9) is performed over the gain medium region. Recall that in the case of PCSELs, which consist of finite-size photonic-crystal slabs, the mode profile can be decomposed into small-scale Bloch mode $u_{\boldsymbol{\Gamma}}(x_0, y_0, z)$ at $\boldsymbol{\Gamma}$ point, and large-scale envelopes $f_i(X, Y)$ that modulate the Bloch mode, namely,

$$\varphi_i(\boldsymbol{r}) = u_{\boldsymbol{\Gamma}}(x_0, y_0, z) f_i(X, Y) \tag{S38}$$

We look for the single-mode steady-state solution of Eq. S35-S37, i.e., $I_{0,ss} > 0$, $I_{1,ss} = 0$, and $\frac{d}{dt}I_{0,ss} = \frac{d}{dt}I_{1,ss} = 0$. Substituting these conditions into Eq. S35, we obtain

$$I_{0,ss} = \frac{g - \alpha_0}{\chi_{00}} > 0 \tag{S39}$$

Therefore, the threshold of the fundamental mode is $g_0 = \alpha_0$. Next, we carry out linear stability analysis around the single-mode steady-state solution to find the threshold of the first-order mode. Assuming the steady state intensity $I_{1,ss}$ is perturbed by a small perturbation δI_1, i.e., $I_1 = I_{1,ss} + \delta I_1$, we can the find the temporal variation of the perturbed intensity up to the first order of δI_1 as

$$\frac{d}{dt}\delta I_1 = 2\delta I_1\left(g - \alpha_1 - \chi_{10}I_{0,ss}\right) \tag{S40}$$

For the single-mode steady state to remain stable, one requires $\frac{d}{dt}\delta I_1 \leq 0$. Therefore, the gain must satisfy the following condition

$$g - \alpha_1 - \chi_{10} I_{0,ss} \leq 0 \tag{S41}$$

Substituting the steady-state intensity of fundament mode $I_{0,ss}$ into Eq. S41, we obtain the threshold of the first-order mode as

$$g_1 = \frac{\alpha_1 - \mathcal{F}\alpha_0}{1 - \mathcal{F}} \tag{S42}$$

Here we define the modal overlap factor $\mathcal{F} = \frac{\chi_{10}}{\chi_{00}}$. The difference between the fundamental and first-mode modes then is given by

$$\Delta g = g_1 - g_0 = \frac{\alpha_1 - \alpha_0}{1 - \mathcal{F}} = \frac{\Delta\alpha}{1 - \mathcal{F}} \tag{S43}$$

4. Suboptimal designs of claddings

In general, a mode with spatially flattened modal profile can be realized by applying cladding structures to the bulk cavity region and fine tuning the structure parameters. However, for the purpose of single-mode lasing, the spatially flattened mode should be the fundamental mode with the lowest radiation loss. Moreover, the surface radiation loss should be comparable to or much larger than the edge radiation loss to ensure efficient surface emission. These conditions require specifically designed claddings. Below, we will show several suboptimal designs of claddings to highlight the superior performance of the spatially varying claddings in our quasi-flat cavity.

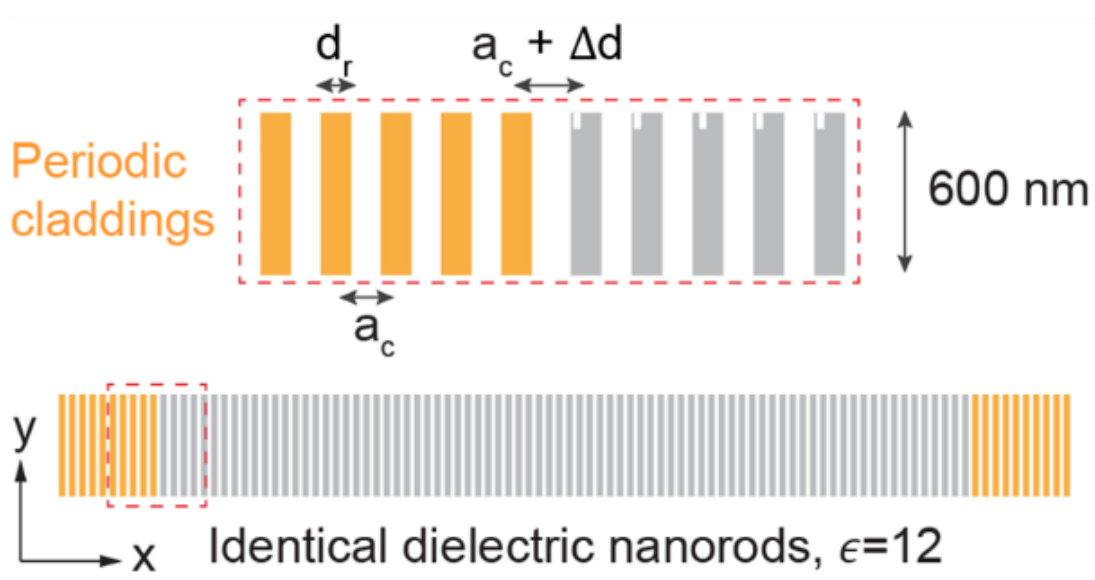

Cladding design	a_c	d_r	Δd
DBR	455.5 nm	102 nm	-165.7 nm
PhC 1	500 nm	282.5 nm	0.6 nm
PhC 2	500 nm	292.5 nm	-29.3 nm

Figure S3. Schematic of periodic claddings (left) and 3 different designs with corresponding parameters. Three different designs are considered. DBR: distributed Bragg reflector; PhC: photonic crystal.

We consider three different designs of claddings that consist of spatially uniform structures. One design is based on the distributed Bragg reflector (DBR), which consists of alternating layers of air and dielectric nanorods. For a fair comparison, we consider a bulk region of 100 nanorods and set the number of nanorods in the claddings on both sides to be 20. This is consistent with the number of nanorods considered in Fig. 4. The exact structure parameters of the DBR are provided on the table in Fig. S3. Specifically, the reflection

phase at the bulk-DBR interface is controlled by fine tuning the spacing between the DBR and the bulk region with a tuning parameter Δd.

Figure S4a shows the radiation loss of the eigenmodes (top), the horizontal cuts of the electric field distributions (E_z) of the fundamental and first-order modes (middle), and the two-dimensional electric field distribution of the fundamental mode (bottom). By fine tuning the bulk-DBR spacing to $\Delta d = -165.7\ nm$, the modal profile of the fundamental mode becomes spatially uniform in the bulk region. However, the bulk-DBR interface exhibits significant diffraction due to the abrupt change in structures. The radiation loss of the fundamental mode is almost two orders of magnitude higher than the surface radiation loss, which can be approximated by $\gamma_{s,\Gamma} = 2.2 \times 10^8\ rad/s$, the radiation constant of the $\gamma_s(\boldsymbol{k})$ dispersion at $\boldsymbol{k} = 0$. In contrast, the radiation loss of the fundamental mode in our quasi-flat structure (orange triangles) is as low as $6.48 \times 10^8\ rad/s$, two orders of magnitude smaller than the radiation loss of the fundamental mode in the DBR design.

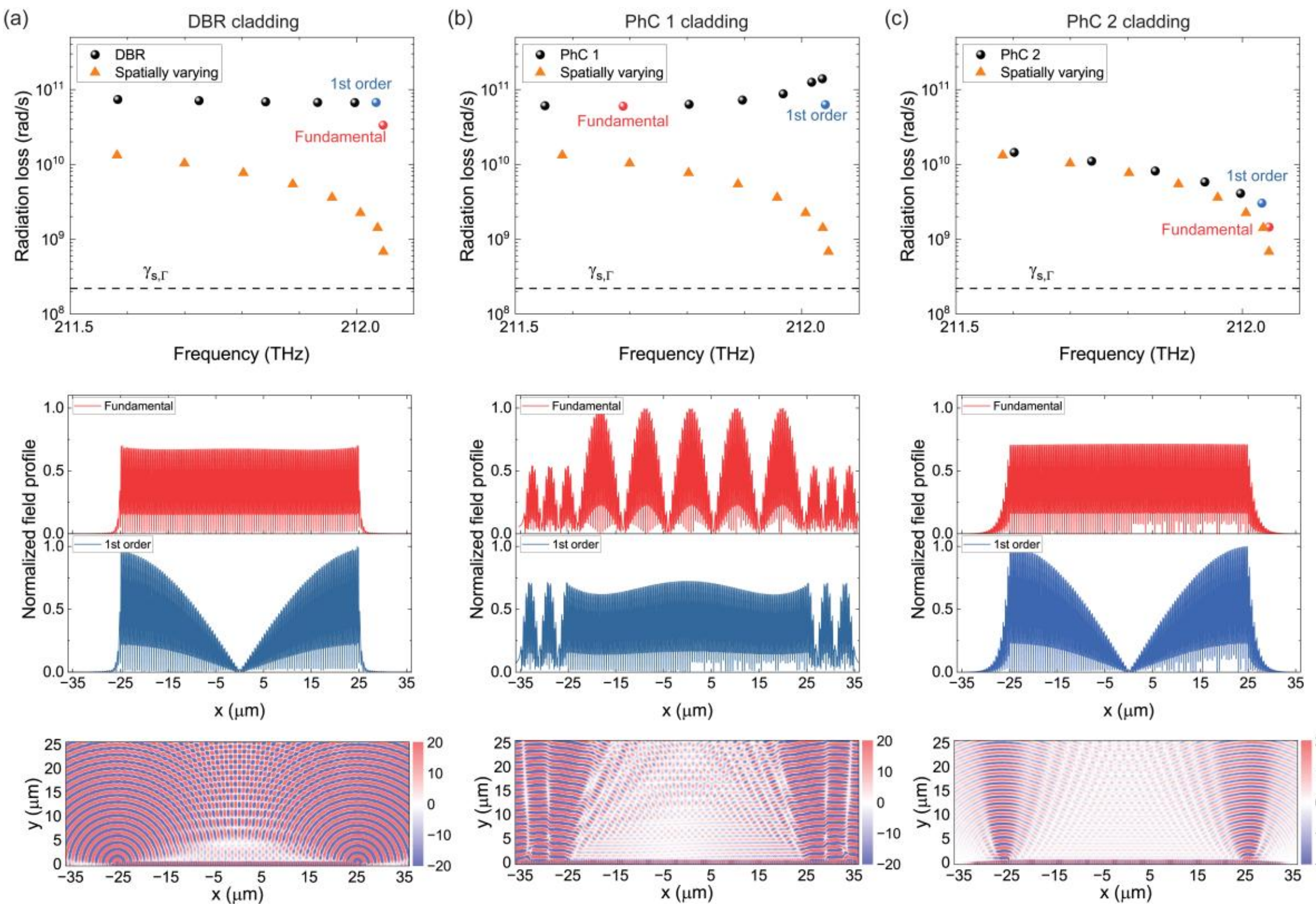

Fig. S4. Three different designs of spatially uniform claddings that are not optimal for lasing, the DBR cladding (a) and two claddings based on photonic crystals (b-c). The DBR cladding can achieve a fundamental mode with spatially flat profile but exhibits significant edge radiation loss. In the PhC 1 design, waves can propagate in the claddings and form standing waves. This design can flatten the profile of the mode with highest frequency, which is the mode intended for single-mode lasing. However, this mode is no longer the mode with lowest loss. Furthermore, the profile of the "1st order" mode is not exactly flat. In contrast, there are only exponentially decaying waves in the claddings in the PhC 2 design. This design achieves the desired fundamental mode with flat modal profile. The edge radiation loss is reduced but is still more than two times larger than our quasi-flat design with spatially varying claddings. Top row: radiation loss of the modes. Middle: horizontal cuts of the electric field (E_z) distributions at $y = 0$. Bottom: two-dimensional electric field distributions.

We then consider a cladding design that has similar photonic crystal structures with the bulk region. Specifically, the lattice constant is the same in both the claddings and the bulk region, i.e., 500 nm. The width of the nanorods is reduced from 287.5 nm in the bulk region to 282.5 nm in the cladding region. To reduce surface scattering in the cladding region, the smaller air void is removed for unit cells in the claddings. The corresponding band edge frequencies are 212.05 THz and 214.05 THz for the unit cells in the bulk region and the claddings, respectively. This configuration allows waves from the bulk region to propagate along the claddings and should reduce the scattering at the bulk-cladding interface. By fine tuning the spacing between the claddings and the bulk, the profile of the first-order mode can be flattened as shown in Fig. S4b. However, this mode is no longer the mode with the lowest loss. Moreover, while the scattering at the interface is reduced, the propagating waves in the claddings form standing waves with strong amplitude, resulting in strong surface scattering for the first-order mode.

Next, we consider a different design based on photonic crystals, which we refer to as the PhC 2 design. A similar design has been considered and demonstrated in Ref. [3]. The width of the nanorods in the claddings is increased to 292.5 nm and the corresponding band edge frequency of the unit cell in the claddings is 210.1 THz. This band structure creates a band gap above 210.1 THz. Consequently, the waves in the bulk region exponentially decay into the claddings, as shown in Fig. S4c. Both the scattering loss at the bulk-cladding interface and the scattering loss within the claddings are reduced. As such, a fundamental mode with spatially flat profile and lowest radiation loss is achieved as shown in Fig. S4c. The radiation loss of the fundamental loss is reduced to $1.45 \times 10^9\ rad/s$, which is still almost 7 times higher than $\gamma_{s,\Gamma}$ and 2.5 times higher than that of our quasi-flat design. As a result, the emission of the fundamental mode is still dominated by the edge scattering as shown in Fig. S4c. Similar behavior was observed in Ref. [3], where the flat mode is also achieved by making the claddings as a band gap for the modes in the bulk region.

5. Far-field profile of larger photonic-crystal slab cavities

Here we numerically demonstrate the far-field profiles of our quasi-flat cavities with different sizes for the bulk region. It is important to note that full-wave simulation of large-area photonic-crystal slab cavities is extremely computationally intensive and is even beyond the capabilities of commercial facilities. Therefore, we use our EFA formalism and Fourier transform to estimate the far-field profile of the fundamental mode.

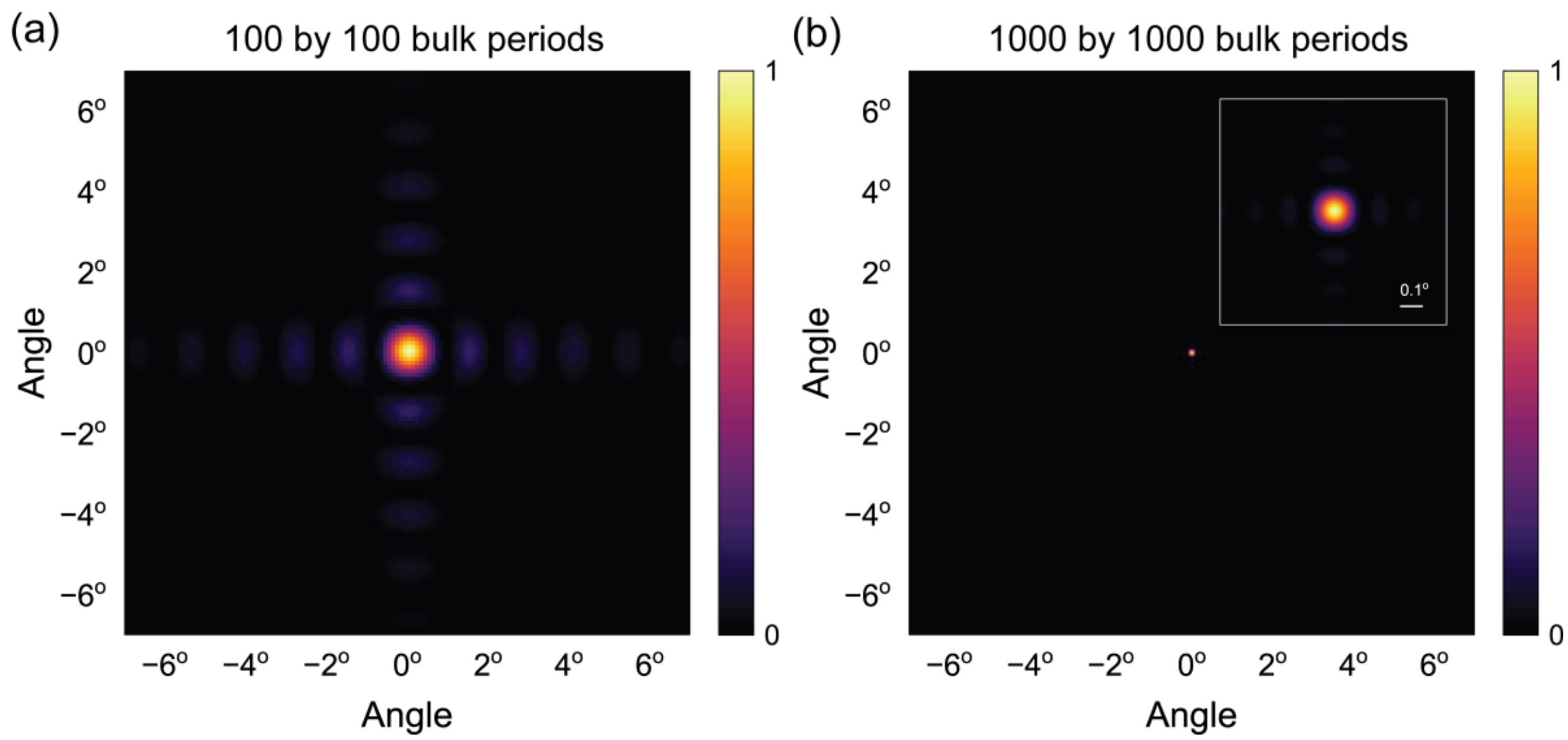

Fig. S5. Normalized far-field profiles of two quasi-flat cavities with (a) 100 by 100 bulk periods and (b) 1000 by 1000 bulk periods.

Figure S5 shows the far-field profiles of the fundamental modes in two quasi-flat cavities with different sizes for the bulk region. For the cavity with 100 by 100 bulk periods, the far-field profile calculated using EFA agrees well with that from full-wave simulations in Fig. 6. When the size of the bulk region is increased to 1000 by 1000 periods, the side lobes are significantly reduced, and the divergence angle is also reduced to ~0.1°.

6. Effect of disorders due to fabrication imperfections

Here we provide a qualitative analysis of the effects of fabrication imperfections on the modal properties of our quasi-flat cavity.

Localization length in disordered photonic crystal. We adopt the optimal fluctuation theory[4] with our envelope function approximation (EFA) to qualitatively analyze the dependence of localization length on the photonic band structure. For simplicity, we consider a quadratic band edge to elucidate the underlying principle. The formalism can be extended to linear dispersion or more complex dispersions[5], but it requires extensive numerical efforts as closed-form analytical solutions typically do not exist.

We assume the photonic band structure near the Brillouin zone center Γ can be described as

$$\Omega(k) = \Omega_0 + A|\boldsymbol{k}|^2 \tag{S44}$$

Here ω_0 and A are the band edge frequency and curvature, respectively. In the weak disorder regime, the corresponding envelope function equation then can be written as

$$-A\nabla^2 f(x,y) + [\Omega_0 + \Delta\Omega(x,y)]f(x,y) = \Omega f(x,y) \tag{S45}$$

where $f(x,y)$ is the envelop function of the modes. $\Delta\Omega(x,y)$ describes the random fluctuation of the band edge frequency of each unit cell. Assuming the random fluctuation

satisfies normal distribution, the disorder strength then can be described by the spatial variance σ_{Ω_0} of the band edge frequency $\Omega_0 + \Delta\Omega(x, y)$.

The above formulism allows us to qualitatively analyze the spatial localization of the modes in disordered photonic crystals using the optimal fluctuation theory. Within a given area L_{local}^2, the disorder functions as a random potential with a potential depth given by

$$\Omega_{rms} = \Omega_0 \pm \frac{\sigma_{\Omega_0}}{L_{local}} \tag{S46}$$

Here we use the fact that the disorder is statistically symmetric around Ω_0, and the probability of the disorder creating a potential well of depth $+\sigma/L_{local}$ is the same as it creating a well of depth $-\sigma/L_{local}$ Within the box of side length L_{local}, the energy of the fundamental mode can be approximated as

$$E_0 \approx \Omega_0 + \frac{A}{L_{local}^2} \tag{S47}$$

Note, we have assumed that $\hbar = 1$ for compactness.

To trap the fundamental mode within the area of L_{local}^2, the energy of the fundamental mode needs to be within the depth of the potential well, which leads to

$$\frac{|A|}{L_{local}^2} \approx \frac{\sigma_{\Omega_0}}{L_{local}} \tag{S48}$$

The localization length then can be approximated as

$$L_{local} \approx \frac{|A|}{\sigma_{\Omega_0}} \tag{S49}$$

Therefore, the localization length is proportional to the band curvature $|A|$ of a quadratic band edge.

Similar analysis and arguments can also be applied to linear dispersions. In the case of linear dispersions, the energy of the fundamental mode can be approximated as

$$E_0 \approx \Omega_0 + \frac{A_{linear}}{L_{local}} \tag{S50}$$

For start-of-art photonic crystal cavities, the fabrication disorders are minimal, and the spatial variance is small compared to the band curvature $\sigma_{\Omega_0} \ll |A_{linear}|$. Therefore, the energy of the fundamental mode exceeds the depth of the potential well and is spatially extended across the entire cavity. In other words, linear dispersion provides much longer localization length than quadratic dispersion.

Effects of disorder on the quasi-flat cavity. Next, we analyze the effect of disorders on the modal profiles in the quasi-flat cavity. For consistency, we consider the same unit cell design in Fig. 6 in the manuscript. It consists of square lattices of circular air holes in a

dielectric slab with a refractive index of 3.491 and a thickness of 260nm. The radius of the air holes is $r_0 = 98nm$, and the symmetry of the airhole is broken by introducing a small circular perturbation with a radius of 30 nm. The lattice constant in the bulk region is $a_0 = 309\ nm$. The corresponding band structure (Fig. S6a) exhibits a quadratic band edge around Γ, with a band edge frequency of $\Omega_0 = 336.04\ THz$.

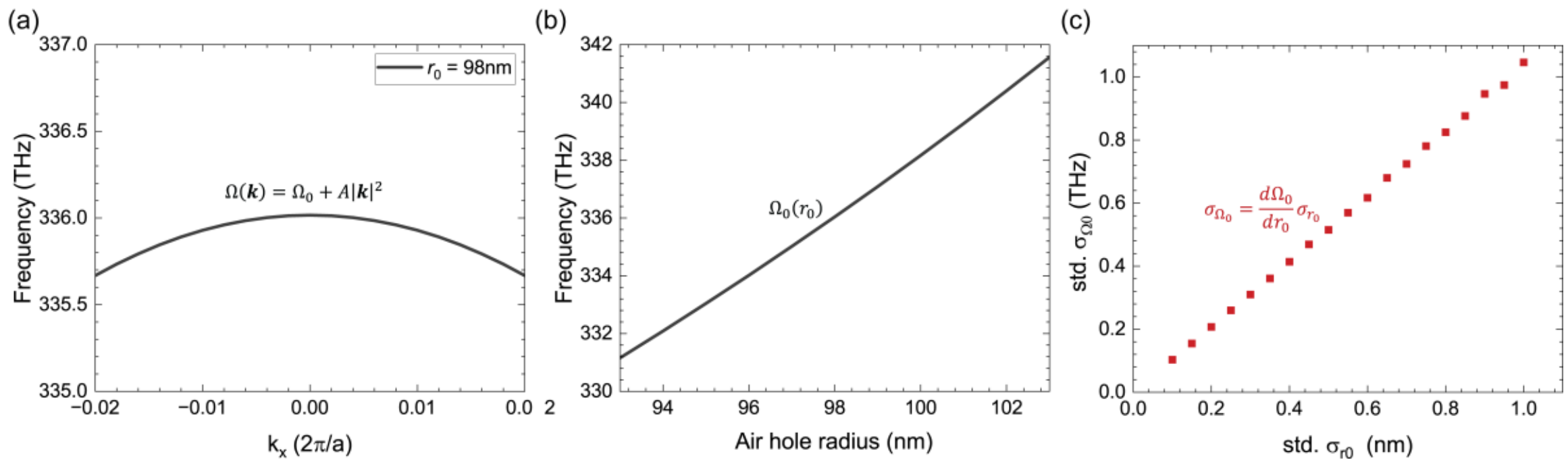

Figure S6. (a) Band structure of the infinitely periodic photonic crystal slab corresponding to the structure in Fig. 6 in the main text. (b) Band edge frequency as a function of the air hole radius. (c) The corresponding spatial variance σ_{Ω_0} of band edge frequencies in the presence of random variations in the air hole radius r_0 with spatial variance of σ_{r_0}.

In practice, the band edge frequency will shift due to fabrication imperfections. Here we assume the radius r_0 of each unit cell is randomly perturbed, and the variation satisfies normal distribution. The disorder strength then is quantified by the spatial variance σ_{r_0}. Throughout our calculation, we use $\sigma_{r_0} = 0.25nm$, which is consistent with the state-of-the-art demonstration in Ref. [6].

To extract the spatial variance σ_{Ω_0} of the band edge frequency, we sweep the radius r_0 of the air hole and calculate the corresponding band edge frequency Ω_0. Figure S6b shows the band edge frequency $\Omega_0$as a function of r_0, which exhibits linear relation. By extracting the slope $\frac{\partial\Omega_0}{\partial r_0}$, one can then find σ_{Ω_0} as $\sigma_{\Omega_0} = \frac{\partial\Omega_0}{\partial r_0}\sigma_{r_0}$. The localization length then can be approximated as

$$L_{local} \approx \frac{|A|}{\frac{d\Omega_0}{dr_0}\sigma_{r_0}} \tag{S51}$$

Therefore, by using the band curvature A and the slope $\frac{\partial\Omega_0}{\partial r_0}$, we find that the localization length $L_{local} \approx 170a_0$ for the specific photonic crystal structure considered here.

Finally, we analyze the effect of disorders on the mode profile of the fundamental quasi-flat mode. Given the localization length of $L_{local} \approx 170a_0$, we choose three different sizes of bulk region: $L_{bulk} = 100a_0, 170a_0$ and $300a_0$. For each bulk size, three different random seeds are used to create different random disorders. The mode profile of the fundamental mode then is calculated using the envelope function approach.

To clearly illustrate the effect of disorders, we first consider the case where the disorder is only applied to the bulk region. Fig. S7 shows the field profiles of the fundamental mode with different bulk sizes. As expected, when the bulk size is smaller than the localization length (left), the mode profile within the bulk region remains relatively uniform. As the size of the bulk region increases to reach above the localization length ($170a_0$), spatial localization emerges.

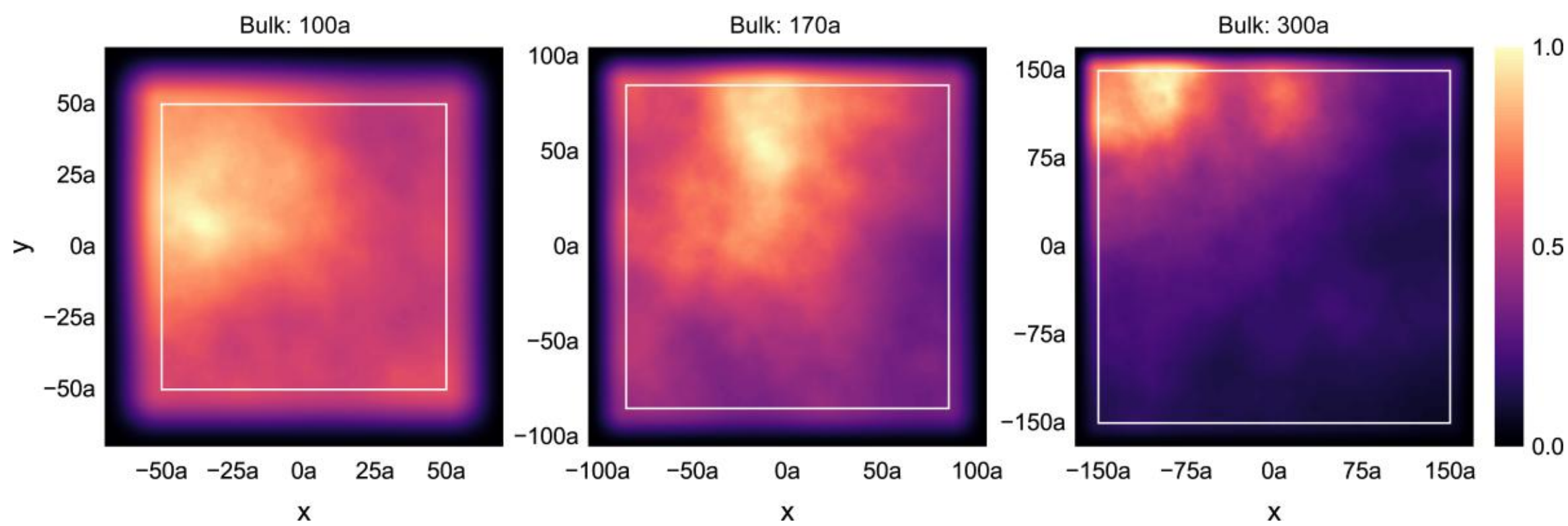

Figure S7. Mode profiles of the fundamental mode when the disorder is only applied to the bulk region.

Next, we consider the case where the disorder is applied only to the cladding region. Fig. S8 shows the field profiles of the fundamental mode with different bulk sizes. Because the cladding region is only 20-periods thick, which is much shorter than the localization length of $170a_0$, the reflection phase at the bulk-cladding interface is relatively insensitive to the disorder in the cladding region. As a result, the field profile within the bulk region remains almost uniform, even when the bulk size is larger than the localization length.

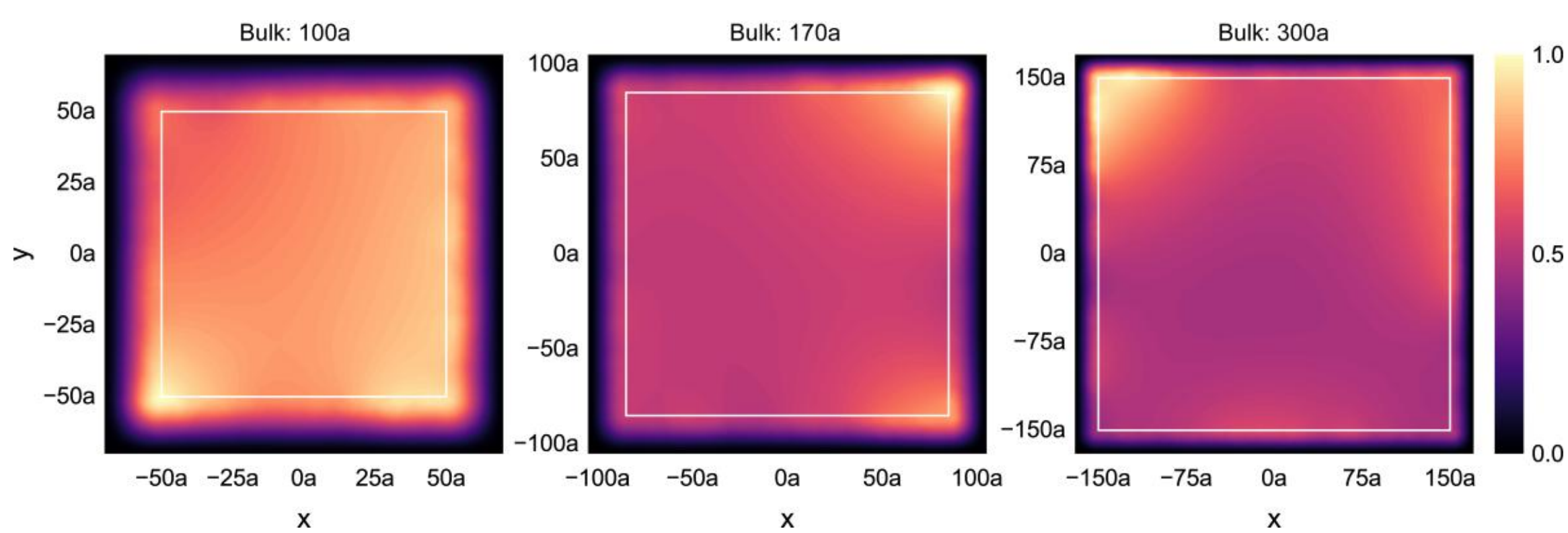

Figure S8. Mode profiles of the fundamental mode when the disorder is only applied to the cladding region.

References

1 de Sterke CM, Sipe JE. Envelope-function approach for the electrodynamics of nonlinear periodic structures. *Phys Rev A* 1988; **38**: 5149–5165.

2 Sipe JE. Vector $k\ensuremath{\cdot}p$ approach for photonic band structures. *Phys Rev E* 2000; **62**: 5672–5677.

3 Li P, Zhang X, Wang F, Chen Y, Yin X, Peng C. Wave morphing and flat-top ground states in photonics systems driven by artificial gauge fields. 2025. doi:10.48550/arXiv.2509.21183.

4 Mirlin AD. Statistics of energy levels and eigenfunctions in disordered systems. *Physics Reports* 2000; **326**: 259–382.

5 Sipe JE, Bhat NAR, Chak P, Pereira S. Effective field theory for the nonlinear optical properties of photonic crystals. *Phys Rev E* 2004; **69**: 016604.

6 Asano T, Ochi Y, Takahashi Y, Kishimoto K, Noda S. Photonic crystal nanocavity with a Q factor exceeding eleven million. *Opt Express, OE* 2017; **25**: 1769–1777.
